# Toponym-assisted map georeferencing: Evaluating the use of toponyms for the digitization of map collections

**DOI:** 10.1371/journal.pone.0260039

**Published:** 2021-11-18

**Authors:** Karim Bahgat, Dan Runfola

**Affiliations:** Department of Applied Science, William & Mary, Williamsburg, VA, United States of America; Universidade Federal de Uberlandia, BRAZIL

## Abstract

A great deal of information is contained within archival maps—ranging from historic political boundaries, to mineral resources, to the locations of cultural landmarks. There are many ongoing efforts to preserve and digitize historic maps so that the information contained within them can be stored and analyzed efficiently. A major barrier to such map digitizing efforts is that the geographic location of each map is typically unknown and must be determined through an often slow and manual process known as georeferencing. To mitigate the time costs associated with the georeferencing process, this paper introduces a fully automated method based on map toponym (place name) labels. It is the first study to demonstrate these methods across a wide range of both simulated and real-world maps. We find that toponym-based georeferencing is sufficiently accurate to be used for data extraction purposes in nearly half of all cases. We make our implementation available to the wider research community through fully open-source replication code, as well as an online georeferencing tool, and highlight areas of improvement for future research. It is hoped that the practical implications of this research will allow for larger and more efficient processing and digitizing of map information for researchers, institutions, and the general public.

## Introduction

Institutions across the globe are actively digitizing and georeferencing collections of physical (or printed) maps [1–9], enabling the information within them to be searched, discovered, and otherwise accessed using contemporary tools [10–14]. However, the technologies and practices of georeferencing in use today have remained largely unchanged since the 1980s [15]. These practices—frequently involving human operators identifying sets of corresponding points [16–19] —represent a significant bottleneck for governments, libraries, and other entities that seek to provide geographically query-able data based on archival maps (c.f. [20–22]).

As an illustrative example of the scope of this challenge, data from the crowdsourced georeferencing website MapWarper [22] shows that between the years 2015 and 2019, online contributors produced approximately 19,000 manually georeferenced maps— spending an average of 92 minutes on each map. With over 714 known map collections held by libraries, archives, and museums globally, many holding hundreds of thousands of maps [23], such manual processes are insufficient to enable broad-scope access to cartographic information in the face of constraints on time and resources.

A number of approaches have been pursued to mitigate the costs of georeferencing. Most common are solutions designed to improve the human experience of georeferencing, seeking to minimize time costs by providing better software environments [10, 12, 19, 20, 22, 24, 25]. A more limited number of approaches have begun to emerge that seek to automate parts of this process, using information on maps (for example, grid-lines or coordinate labels) to make automated attempts at georeferencing [20, 26].

In this study we build on these automated approaches, specifically asking the question: *to what degree can map georeferencing be automated through the use of map toponym labels?* Unlike past approaches, which are reliant on features (grid lines, road lines) that are present on only a fraction of maps, the use of map toponyms would allow for near-universal georeferencing, as nearly all maps have labeled places on them. Towards this end, the goal of this paper is to present the first fully automated toponym-based georeferencing methodology, test the approach using large sets of real and simulated maps, and provision associated code and tools to the public.

The paper is structured as follows. First, we give an account of the current state of the art research on automated georeferencing and highlight issues that have yet to be addressed. Second, we describe our implementation of toponym-assisted map georeferencing, making several contributions in the areas of text recognition, toponym disambiguation, and (dynamic) transform estimation. Third, we conduct a large-scale evaluation of this approach for a range of real and simulated maps to illustrate accuracy and efficiency under different circumstances. Finally, we discuss some of the implications and remaining limitations with the proposed methodology.

For users that are interested in applying the outlined procedures to their own map documents, we have implemented a version of the methodology as part of a free web-based georeferencing tool at: www.maplocate.org.

### Previous research on automated map georeferencing

Georeferencing has been used to digitize the contents of maps into actionable data going back as far as the 1960s [15, 17, 27]. These efforts have enabled maps to be used as source material in cadestral and land-use databases used by local governments, global administrative boundary datasets, databases of fauna and soil distribution, and databases of oil-and-gas exploration, among many other applications [28–33]. As sources of unique historical information, data extracted from maps are frequently used in everything from the study of land-use change and hydrological mapping, to research on international development and political conflict [34–43]. However, the georeferencing process is itself resource intensive, leading researchers to explore methods for full or semi-automation [20, 26].

Researchers have explored at least three approaches for automated (either fully or human assisted) map georeferencing to-date: coordinate-based, feature-based, and toponym-based. First, coordinate-based georeferencing attempts to detect explicitly stated information about the map’s coordinate system. This involves—for example—searching for map grid lines and tick marks that are marked with coordinate labels, or using line-tracing and text recognition to parse and place control points at the grid intersections [20, 21, 25, 44, 45]. Others have used similar techniques to detect labelled map corner coordinates typical for small-extent local maps [26, 37, 46].

Second, in feature-based georeferencing, the goal is to detect one or more thematic feature layers shown in the map (e.g., roads), and then compare that information with some reference data containing known coordinates [47–52]. The primary challenges identified in this literature include, first, how to accurately identify the thematic feature layers, and second, how to efficiently compare complex map feature s with a potentially much larger global reference set (i.e., the problem of point set conflation; [53, 54]).

A third approach is seen in the emerging literature on toponym-based georeferencing. Here, the central idea is to detect toponym map labels, specifically placename toponyms (i.e. the names of cities or towns), and determine a set of control points by matching these to a reference gazetteer dataset containing toponym coordinates [8, 55–58]. In addition to being nearly ubiquitous on maps, toponym labels are some of the most easily identifiable point-like features on a map. Toponyms therefore serve as a natural choice for control point selection. Implemented in a semi-automated workflow, all that is required is for the user to locate placename toponyms on the map and label them [24], which both improves efficiency and lowers the level of skill required. Furthermore, the feasibility of finding matching control point coordinates is helped by the availability of several global gazetteer dictionary sources containing the coordinates of placename toponyms. Lastly, unlike administrative boundaries, roads, rivers, and buildings which may change frequently, the toponyms associated with particular places typically operate as historical markers and remain unchanged over long time periods [59, 60].

Today, the literature on toponym-based georeferencing is largely small scale (i.e., based on anecdotal numbers of maps), and narrow in focus on specific challenges, such as text recognition (c.f., [8, 55–58]). This paper seeks to overcome these limitations, providing a test of the accuracy of a fully-implemented toponym-based georeferencing method across a large set of both real-world and simulated maps.

## Materials and methods

A methodology for automated toponym-assisted georeferencing must address and overcome three major challenges: 1) how to extract a set of toponym labels (and the associated image coordinates of markers) from the map, 2) how to determine the possible geographic coordinates of these labels, and 3) estimating the most appropriate transform function.

### 1) Identifying toponym control points in a map

**Fig 1:**
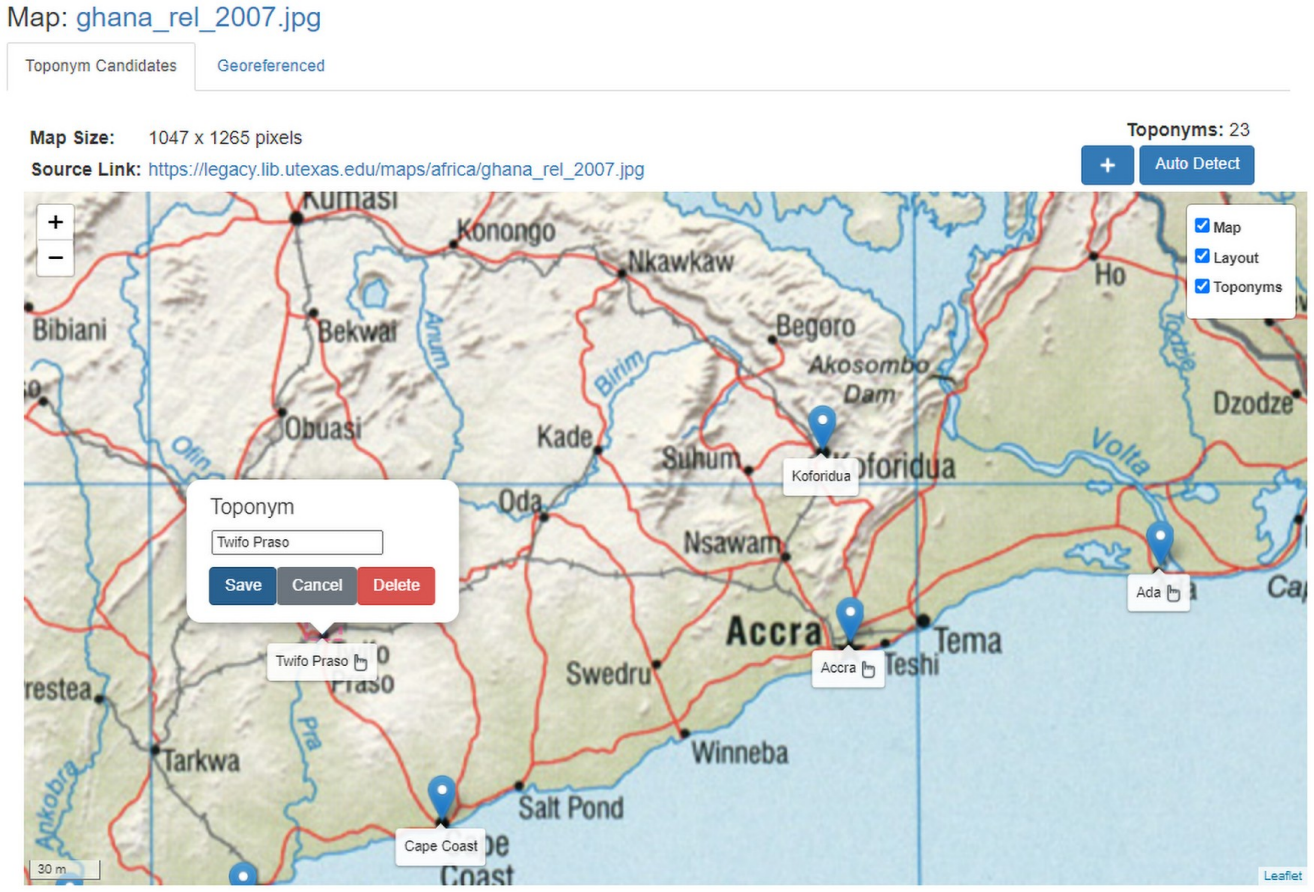
Example of a semi-automated workflow. Screenshot of a toponym-assisted georeferencing workflow. In a semi-automated workflow, if a user notices problems with toponym-based georeferencing, adjustments can be made through the manual selection and correction of control points. The map image is from the University of Texas at Austin’s Perry-Castañeda Library (PCL) Map Collection and is in the public domain.

Since toponym-assisted georeferencing is based on the name and placement of placename toponym labels, the first step is in how to extract these text labels from the map. In a semi-automated workflow, this step could be accomplished through an interactive map interface that lets a user identify and place toponym control points (or adjust existing ones), where all the user needs to provide is a point location and the toponym text associated with that point (see Fig 1).

In a fully automated workflow, the identification of toponyms in maps presents unique challenges compared to traditional text documents, and is an active area of research [5]. With the exception of some early-stage research on map-based OCR engines [58, 61, 62], most approaches break the process into three discrete stages: a) the separation of text pixels from the surrounding background graphics of various colors; b) clustering these pixels to form possible image regions containing letters, words, and multi-word labels, and c) performing text recognition on each pixel cluster. In this paper, we follow a similar three-step approach tailored for the task of toponym extraction.

**Fig 2:**
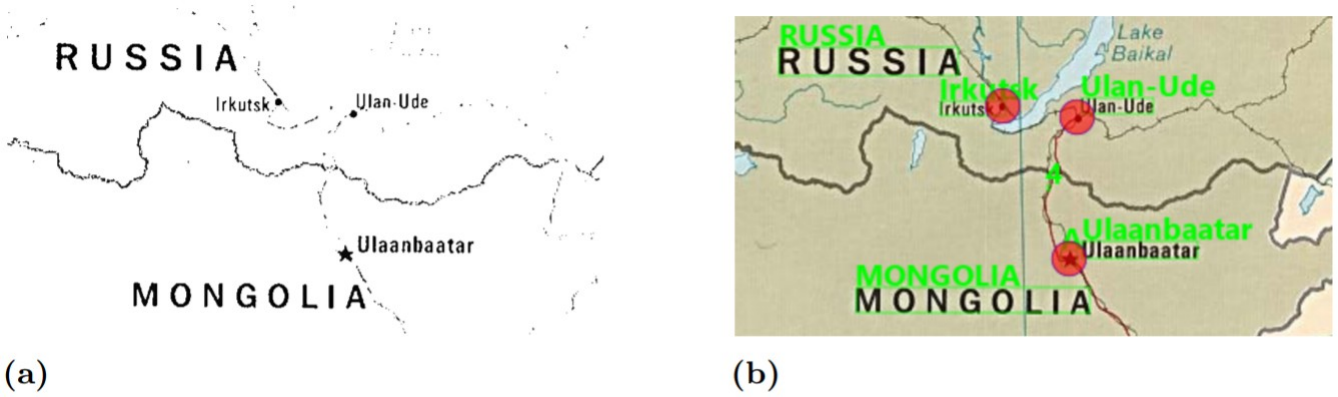
Illustration of toponym recognition. (a) Thresholded image using the △*E* metric for the automated identification of text and toponym markers. (b) Identified map text labels (in green) and toponym marker points (in red). The map image is from the University of Texas at Austin’s Perry-Castañeda Library (PCL) Map Collection and is in the public domain: https://maps.lib.utexas.edu/maps/middle_east_and_asia/china_pol96.jpg.

For the first stage (stage a), we implement a color thresholding approach to define some pixels as “text” and other pixels as “not text”. Contrasted to most existing implementations reliant on grayscale or RGB color thresholding [63–67], our implementation identifies perceived color similarities using the CIELab Delta E 2000 color difference metric △*E* [68]. Since most maps depict toponym labels in some shade of black or dark gray, for our automated approach we choose black as the reference color and isolate those pixels where the fuzzy color difference (△*E*) is smaller than 25 (see Fig 2a)—a hyperparameter that was determined through experimentation in order to capture color variation in edge pixels as well as color distortions in low-resolution images. This approach provides separation of text- from non-text pixels and allows us to skip additional steps such as background line removal [61, 63, 69, 70]. Some of the limitations of this approach, and concomitant future directions for research, are noted in our discussion.

For the second stage (b), rather than identifying individual regions of pixels that make up individual strings of text and processing each separately, we instead apply text recognition to the entire set of text pixels all at once. In the presented work, this process is implemented using the sparse text recognition mode of the popular open-source Tesseract engine, which detects all text throughout the image regardless of font size; more information on this algorithm is available in [71]. To improve handling of pixelated text in low-resolution images, we upscale and resample the image prior to text recognition. This results in a list of identified words and their coordinates, width, height, and confidence level. The text recognition is likely to contain several errors, so we clean the results by dropping text recognized with a low confidence probability (<60%), as well as single-character text, numeric and non-alphabetic characters.

In the third stage (c), we group the identified text to form connected text labels, such as multi-part place names (shown as green rectangles in Fig 2b). We implement a rules-based algorithm that groups similar words within approximately 1.5 font-height distance apart [72]. The result is a list of all text including map titles, legend descriptions, and descriptive sentences. Since we are only interested in text representing toponym labels, we only keep those located within the bounds of the main rectangular map area, and where the first character of each word is capitalized. No rules are implemented with regard to font size (i.e., all font sizes are eligible to be defined as toponyms) to account for maps which may use font size to define toponyms at varying levels of a hierarchy.

Once toponym labels are identified, the coordinates of the symbol associated with a given toponym need to be identified (i.e., the marker—such as a circle or square—representing the toponym’s location on a map). There are many possible approaches to detecting a toponym marker symbol [73–76]; here we implement a contour-based approach that looks for arbitrarily shaped black-colored pixel collections in the neighbourhood of each toponym label. The centroid of the closest such group to each text marker is used as the image coordinate for each toponym (shown as red circles in Fig 2b). Toponyms for which no marker symbol can be detected are removed from the final set.

### 2) Toponym geocoding & disambiguation

In the previous step we identified a set of *N* toponyms: *θ*_*i* = 1_, *θ*_*i* = 2_, *θ*_*i* = …_, *θ*_*i* = *N*_. The next step is to associate each *θ*_*i*_ with their equivalent geographic coordinates by searching gazetteer dictionaries, a process known as geocoding. To allow for flexible usage and wide global coverage, we integrate several publicly available global gazetteers: The USGS Geographic Names Server (GNS) gazetteer [77], the GeoNames gazetteer [78], the CIESIN Global Settlement Points dataset [79], the OpenStreetMap-based placenames dataset [80], and the Natural Earth Populated Places dataset [81]. This database is used to search and lookup the coordinates of the identified toponyms.

**Fig 3:**
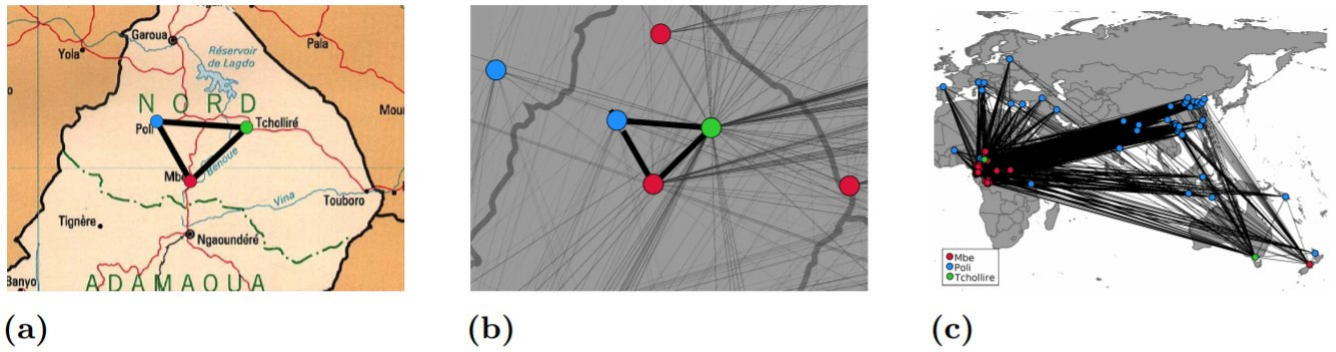
Pattern-based toponym identification. (a) Point pattern of the toponyms Mbe, Poli, and Tchollire detected in the image. (b) Point pattern of the corresponding geographic coordinates to the toponyms. (c) Full view of all candidate gazetteer matches and their point patterns. The map image is from the University of Texas at Austin’s Perry-Castañeda Library (PCL) Map Collection and is in the public domain: https://maps.lib.utexas.edu/maps/africa/cameroon_pol98.jpg. The geodata used to render country outlines is from ©Natural Earth data and is in the public domain.

A major challenge in this step, and with geocoding more broadly, is that toponyms are often ambiguously used in many different parts of the world—i.e. for each toponym *θ*_*i*_ there is typically more than one possible candidate coordinate. Solving this problem requires figuring out which of these candidate coordinates is the correct one, a process known as toponym disambiguation. The problem can be illustrated for the case of three toponyms identified from a map of Cameroon: “Poli”, “Mbe”, and “Tchollire” (Fig 3a). This set of three placename toponyms has thousands of possible matching candidate coordinates (Fig 3c); however, only one of those combinations is the one we are interested in (Fig 3b). To promote a semi- or fully-automated approach to toponym based georeferencing, we must provide an algorithm which serves to disambiguate and find the correct matches among all of these possible coordinates.

Typically, toponym disambiguation is solved by providing additional hierarchical information, such as the country and administrative unit. For map toponyms however, this information is not explicitly given and would have to be inferred manually. Instead, research on toponym-based georeferencing has taken advantage of the fact that we know not just one but multiple toponyms, and that we also have information on their relative spatial locations. Previous approaches in the literature has ranged from simple clustering algorithms to obtain the approximate location of a map [8, 55, 57], to more complicated Bayesian RANSAC probability models [56, 58]. Here, we introduce an alternative approach rooted in the literature on point pattern matching [82–86]. Specifically, we outline an approach based on normalized coordinate space and combinatorial optimization to achieve both efficient and accurate results, which we describe below.

#### Pattern-based toponym disambiguation: Normalizing coordinate space

**Fig 4:**
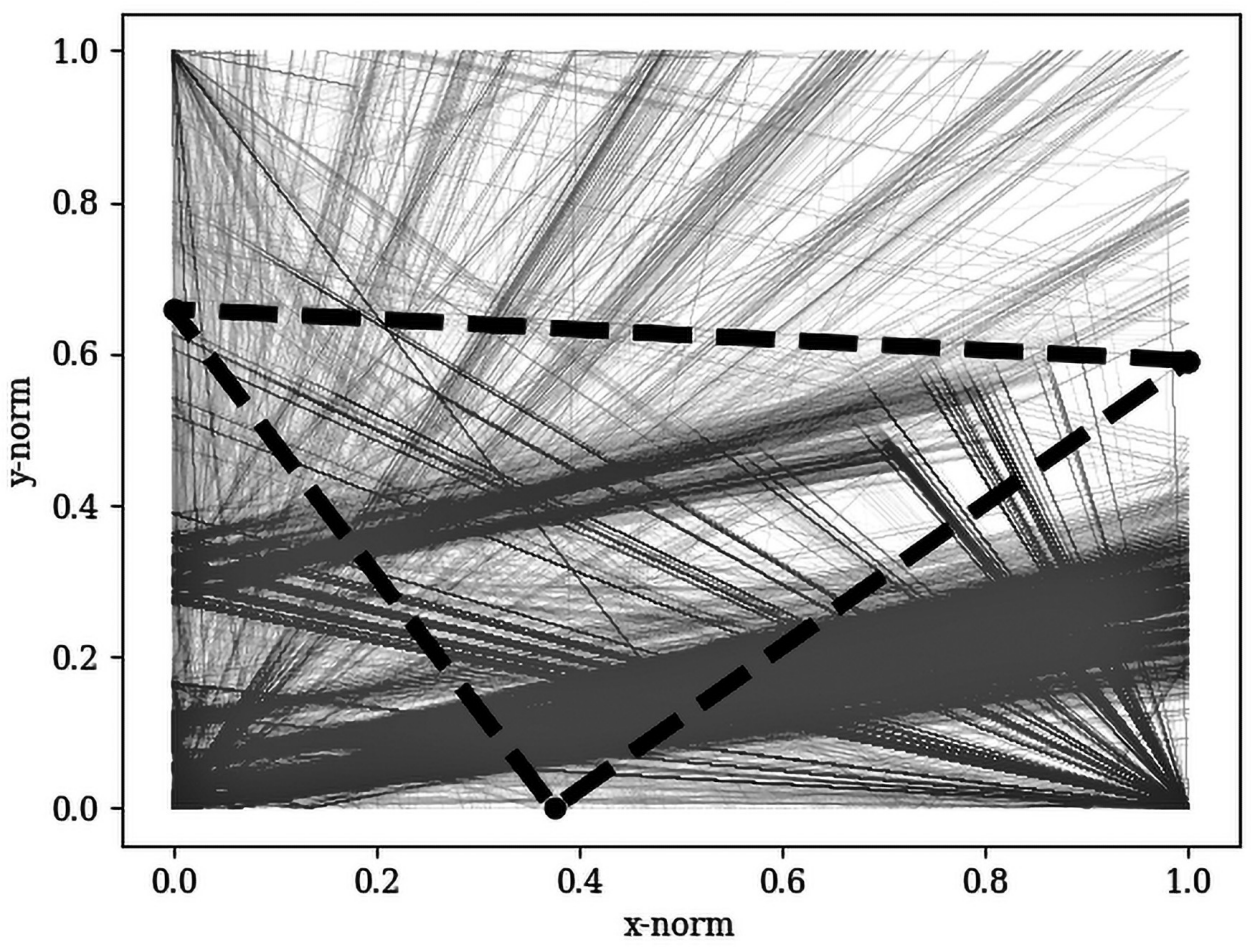
Normalized point pattern coordinates. Pattern matching of image placename toponyms Mbe, Poli, and Tchollire and coordinate combinations in normalized space.

The fundamental idea of using point pattern matching for toponym disambiguation is to identify the pattern that the toponyms make in the un-georeferenced map image, and then compare this pattern to the patterns formed by our georeferenced gazetteer points. For example, if three cities are arranged so as to make an equilateral triangle in the image space, we would seek to find three cities arranged in a similar equilateral triangle in our projected gazetteer space. Before we can compare these point patterns of the image toponyms with their geographic coordinates, which are given in different units (i.e., pixels and decimal degrees), we must first convert them to a common coordinate system. In our approach we normalize their coordinates as values ranging from 0 to 1 between the minimum and maximum x and y coordinates for each point pattern (Fig 4). To preserve the aspect ratio of the point patterns, the longest of the x or y axis will extend to a maximum of 1, while the shortest axis will only range to some fraction of 1 depending on the ratio between the longest and shortest axes. This approach retains the information in the shapes that is most relevant for the algorithm presented here—relative coordinate positions.

#### Pattern-based toponym disambiguation: Match selection procedure

Having normalized all coordinates according to the previous step, consider that the set of original image toponyms forms a pattern *ϕ* in normalized coordinate space. Each of the *θ*_*i*_ toponyms that make up *ϕ* has *M*_*i*_ possible coordinates: ${\hat{\theta }}_{i,j=1},{\hat{\theta }}_{i,j=2},\ldots ,{\hat{\theta }}_{i,j=M_{i}}$. For the full set of toponyms this means there are *Z* possible combinations of all possible ${\hat{\theta }}_{i,j}$ coordinates, where the point pattern of each combination is given as ${\hat{\phi }}_{z}$. Match selection is done by contrasting the original point pattern *ϕ* to the point pattern of each candidate coordinate combination ${\hat{\phi }}_{z}$. We do this following a metric of pattern similarity defined as the average relative distance between coordinates in normalized space:

$$\begin{array}{c} △{\hat{\phi }}_{z}=\sum_{i=1,j}^{N}\sqrt{{\left(x_{i}-{\hat{x}}_{i,j}\right)}^{2}+{\left(y_{i}-{\hat{y}}_{i,j}\right)}^{2}}/N \end{array}$$ (1)

where *N* is the number of toponyms, *x*_*i*_, *y*_*i*_ is the normalized image coordinate for the toponym at *i*, and ${\hat{x}}_{i,j},{\hat{y}}_{i,j}$ is the normalized geographic coordinate of the *j*’th possible match in a particular point pattern combination. All combinations of match candidates that fall below some threshold of similarity *σ*, can be said to resemble the point pattern in the original image while also allowing for map projection distortions and noise from mismatched toponyms. This may result in some incorrect matches for smaller point sets, but the chance of finding multiple parts of the world with the same names and spatial configurations decreases significantly when matching more complex and larger sets of point patterns.

#### Pattern-based toponym disambiguation: Combinatorial optimization

Following this match selection procedure we have narrowed down the list of possible candidate coordinate sets, but still have to decide which of these to use. Comparing all possible matching patterns and choosing the optimal one requires calculating the similarity metric $△{\hat{\phi }}_{z}$ for all possible combinations of coordinates for every toponym. Specifically, the number of combinations for which we would have to calculate the pattern similarity metric is equal to the sum product of the number of possible coordinates of each toponym:

$$\begin{array}{c} Z=\prod_{i=1}^{N}M_{i}=M_{i=0}*M_{i+1}*\ldots *M_{N} \end{array}$$ (2)

where *Z* is the total number of combinations, *M*_*i*_ is the number of possible matches for toponym *θ*_*i*_, and *N* is the number of toponyms. This means that the computational cost grows exponentially as *N* increases. For instance, if there are exactly 5 possible coordinates for every toponym, then the number of comparisons for a mere 10 toponyms is 5^10^ ≈ 10 million. For 20 placenames this increases to 5^20^ ≈ 95 trillion. Most maps will likely have on the order of 30–40 placenames, resulting in an algorithm that would only be practical in a cluster environment if an exhaustive search was used.

To overcome this, we focus on an ordered, piece-wise, growth-based method for quickly finding a number of candidate matching sets (for other approaches, see [87–89]). Our approach is based on the idea that finding a match for just three of the toponyms is often sufficient for finding an initial matching set, and this set can then be expanded iteratively at a much lower computational cost. The presented method consists of five steps:

1. Create a list **T** of all toponyms. From this set of all toponyms **T**, construct a set **C** made up of all possible combinations of three non-repeating toponyms, ordered such that the triplets with the fewest possible toponym coordinates—and therefore least computationally costly to resolve—appear first. For instance, in the case of four toponyms, set **C** would contain three possible combinations:
  $$\begin{array}{c} C=\{\left\{\theta_{1},\theta_{2},\theta_{3}\right\},\left\{\theta_{1},\theta_{2},\theta_{4}\right\},\left\{\theta_{2},\theta_{3},\theta_{4}\right\}\} \end{array}$$ (3)
2. Select and remove the first triplet from set **C**. The goal is then to identify the optimal geographic coordinates—as described in the previous section—for these three toponyms, i.e. the combination of matched toponyms *θ*_*i*,*j*_ whose coordinates best match the original 3-point pattern in the image. The number of comparisons required to find the optimal set of coordinates for these three toponyms is:
  $$\begin{array}{c} Z_{triplet}=\prod_{k=1}^{3}M_{k} \end{array}$$ (4)
  where *M*_*k*_ is the number of possible matches for each toponym *θ*_*k*_ at position *k* in the triplet. If the optimal combination of coordinates for the triplet satisfies some minimum threshold of similarity *σ*, we add the triplet to a result set **R** and proceed to step 3. If not, we repeat step 2 with the next triplet of **C**.
3. We then consider any of the remaining toponyms *θ*_*i*_ in set **T** that have not yet been added to the result set **R**, and determine its geographic coordinate based on the match candidate ${\hat{\theta }}_{i,j}$ that best improves the pattern similarity score $△{\hat{\phi }}_{z}$ of **R**. If the new similarity score for the optimal coordinate remains under the similarity threshold *σ*, we add this toponym to the result set **R**—e.g. updating it from the original 3-point triplet to a 4-point set.
4. Repeat step 3 until all—or as many as possible—toponyms *θ*_*i*_ in set **T** are associated with a matching toponym ${\hat{\theta }}_{i,j}$ in the result set **R**. The remaining number of comparisons required to expand the initial result triplet **R** with all the remaining elements in **T** is then only:
  $$\begin{array}{c} Z_{expand}=\sum_{i=1}^{N-3}M_{i} \end{array}$$ (5)
  where *N* is the number of toponyms, and *M*_*i*_ is the number of possible matches for each toponym *θ*_*i*_ that remains. The expanded result set **R** can be used as the basis for the control points.
5. Since the result set **R** may in rare cases be a spurious match, repeat steps 2 to 4 a given number of times *τ* by looking for additional triplets in **C** that can be matched and expanded, resulting in multiple possible **R** sets of control points. The algorithm ends as soon as the number of matching triplets reaches *τ*, or the total number of comparisons has reached some maximum threshold *ω* (to avoid the cost of a full exhaustive search).

This approach is not guaranteed to find all matching sets—in contrast to a full exhaustive search which would iterate through all possible combinations of place name coordinates. However, it drastically reduces the computational cost of the search while still finding a list of possible matching control point sets that meet a minimum level of similarity. This leaves us with a list of possible sets of control points, with a final stage requiring the selection of a single set of the most optimal control points.

### 3) Transform estimation and selection

To select between the multiple sets of possible control points from the previous step, we use the point correspondences of each set of identified control points to estimate the optimal transformation model for each map image, and then compare and contrast their model fits. The purpose of the transform function in map georeferencing is to translate between image and geographic coordinates in a way that mimics the mathematical equations underlying the original map projection. For the application presented in this paper, we compare the transform functions of the most commonly used 1st, 2nd, and 3rd order polynomial transforms.

To compare and find the optimal transform function we need to measure the accuracy of each set of candidates, which is typically done using the root mean square error (RMSE) of the control point residuals [17]. However, to mitigate overfitting and underreporting of errors in RMSE, as well as a bias towards higher-order polynomials [16, 17, 90, 91], we use an accuracy metric based on out-of-sample or leave-one-out residuals [17, 92, 93], and use the maximum residual for a more conservative estimate, *modelMax*_*LOO*_. Because our sets of control points are likely to contain misidentified outlier points, we also need a way to identify and drop these outliers without the manual input that is typical of traditional map georeferencing [17]. In this paper we implement an automated procedure where we go through each of the control points and estimate the model error *modelMax*_*LOO*_ that would result if that point were to be dropped. The point whose exclusion best improves the model (results in the lowest model error) is then dropped. This is repeated until the model stops improving beyond a specified percentage threshold (e.g. drops below 10%).

Based on these automated procedures we are able to exclude possible outliers and estimate the best model for each set of control points. Having estimated all the candidate models, we can use the *modelMax*_*LOO*_ accuracy metric as the basis for comparing and selecting the set of control points whose transform model achieves the highest accuracy. This step completes the proposed method and gives us the final output: a transformed georeferenced image based on a toponym-assisted process that requires no manual intervention.

### Evaluating the accuracy of toponym assisted georeferencing

In order to evaluate the accuracy of toponym-assisted georeferencing, we engage in two exercises. First, we test our implementation on a large collection of simulated maps and report the accuracy achieved for different types of map parameters. Second, we explore the use and accuracy of the approach for a selection of real-world maps. Overall, the goal is to evaluate and demonstrate that toponym-assisted georeferencing is viable as a general-purpose approach applicable and easily implemented for a large variety of maps.

#### Evaluating georeferencing accuracy for simulated maps

In order to evaluate the accuracy of the toponym-assisted approach for map georeferencing, we first conduct a series of tests on computer simulated maps. This simulation approach a) allows the calculation of the true error of the georeferencing process, and b) provides full control over the test map characteristics, which can be used to evaluate how effective the automated georeferencing is for different types of maps.

**Fig 5:**
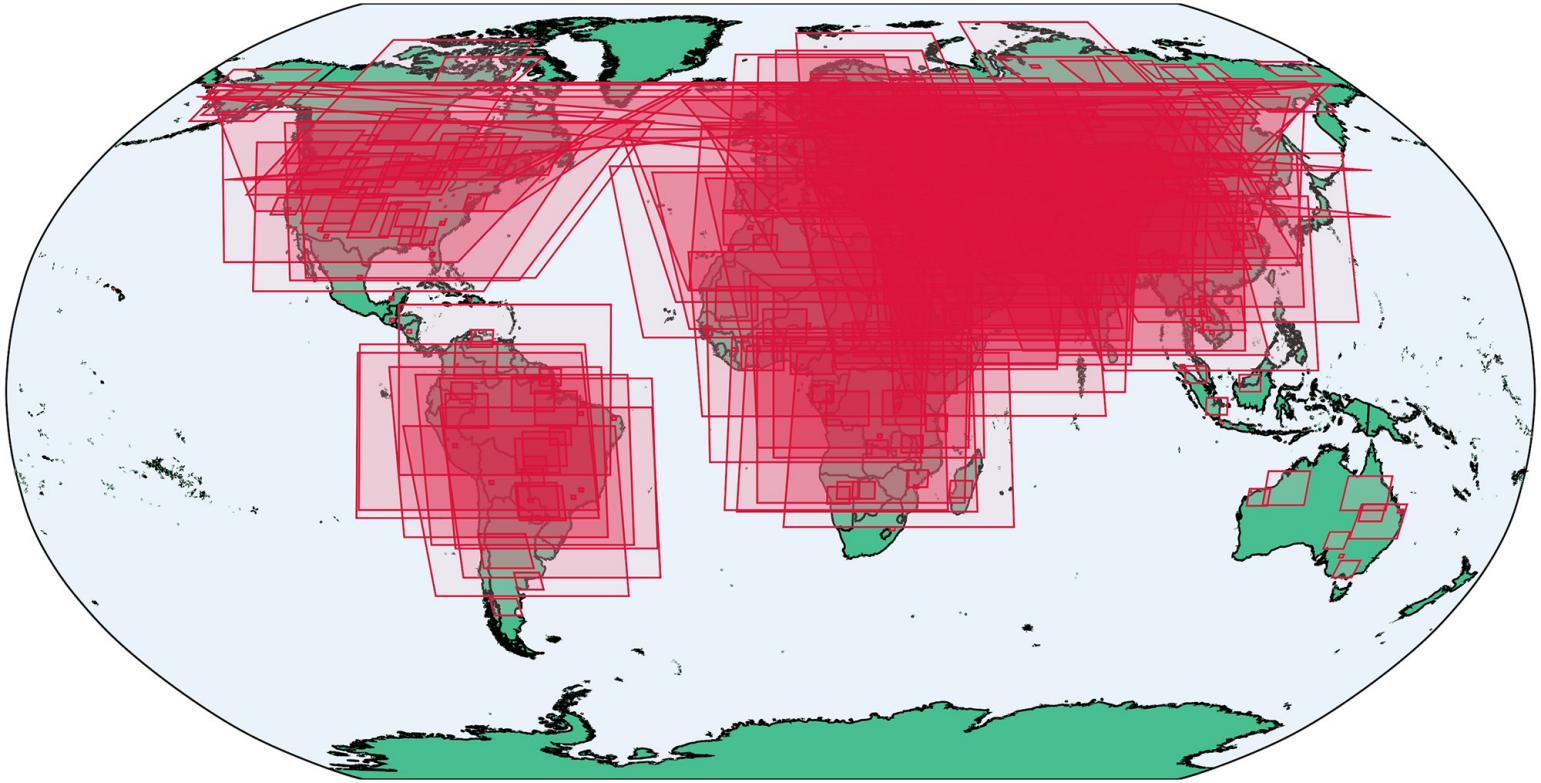
Bounding boxes of the simulated map scenes. The geodata used to render country outlines is from ©Natural Earth data and is in the public domain.

**Fig 6:**
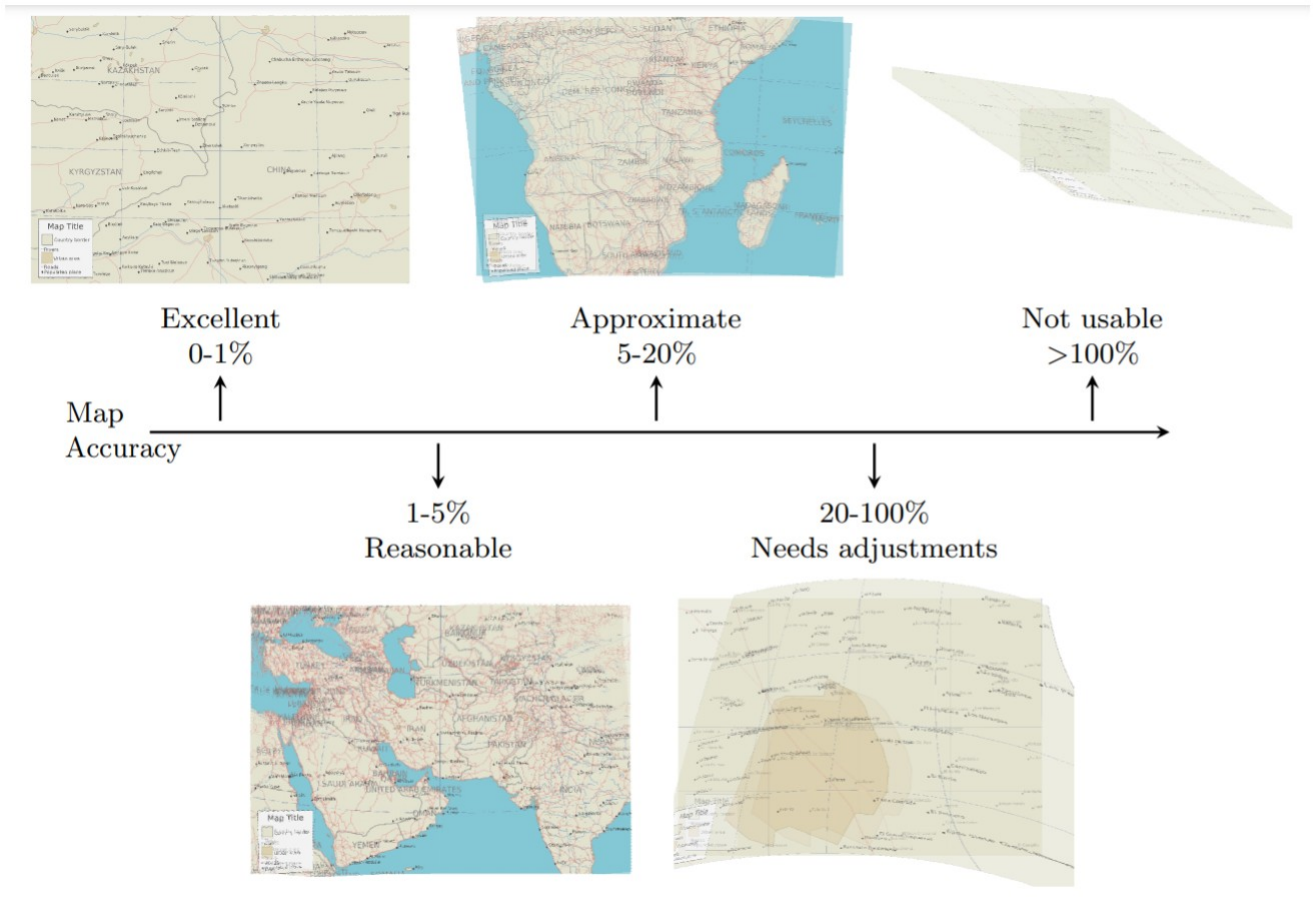
Simulated map accuracy categories. Shows example georeferenced maps overlaid on source maps with known coordinates. Map accuracy is calculated based on normalized maximum map error (as a percentage of image radius). The simulated maps were generated based on public domain data from ©Natural Earth, including data on country outlines, populated places, urban extents, rivers, and roads.

For the map generation process, we selected 379 geographic areas sampled from around the world for which we generate our map simulations (see Fig 5). These “scenes” were defined to ensure a broad range of geographic coverage for primarily land-based locations, with variable numbers of toponyms, map projections, and spatial extents. Each scene was rendered multiple times for different combinations of a set of map parameters: toponym location uncertainty (based on random coordinate offsets), map resolution (defined as the pixel width of the image), and image pixel noise (resulting from lossy image file formats). The map parameters and their values are outlined in Table 1, and were chosen to represent the diversity of maps likely to be encountered in the wild (as well as some more extreme outlier scenarios). In total, 7,580 simulated maps were generated. The simulated maps were rendered using data from Natural Earth [94], including layers for country boundaries, rivers, roads, and urban extents, as well as a map title, legend, and coordinate grid lines (some examples of the simulated maps can be seen in Fig 6).

**Table 1:** Sample sizes of the simulated map parameters.

Parameter	Value	Test maps
**Scene selection:**		
*mapCenter*	Random lat-long coordinate	7,580
*mapExtent*	5000 km	1480
	1000 km	1520
	500 km	1440
	100 km	1680
	25 km	1460
*mapProjection*	Equirectangular	2480
	Lamber Conformal Conic	2540
	Transverse Mercator	2560
*numToponyms*	80 text labels	2060
	40 text labels	2100
	20 text labels	2100
	10 text labels	1320
**Scene permutations:**		
*toponymUncertainty*	0	1895
	1 km	1895
	10 km	1895
	50 km	1895
*imgResolution*	3000 pixels	3032
	2000 pixels	3032
	1000 pixels	1516*
*pixelNoise*	png	4548
	jpg	3032*

* Maps rendered at the coarsest resolution (1000 pixel width) with the noisiest image file format (jpg) were dropped from the analysis, since text labels were illegible for this combination of parameter values.

To evaluate the georeferencing error at each pixel, we leverage the known transform of the map renderer to arrive at the true relationship between pixel and geographic coordinates in the computer generated maps. The georeferencing error of our approach can thus be calculated as the pixel distance from the estimated georeferenced coordinate to the original geographic coordinate for any given point (as opposed to only at the control points). For this exercise we calculate the total map error as the true maximum of all pixel errors, *trueMax*, which constitutes a very conservative accuracy estimate and a more demanding evaluation. To enable the comparison of different map resolutions in this section, we express the maximum error metric in scale independent units by normalizing the error as a percentage of the map radius, i.e. the number of pixels from the center of the map to any of its corners [92, 95]. For example, a normalized maximum error metric of 50% would mean a pixel displacement from one of the corners of the map to about halfway towards the map center. Based on this normalized metric, we subset our results into categories qualitatively representative of the usefulness of toponym-assisted georeferencing under different circumstances: *Excellent*, *Reasonable*, *Approximate*, *Needs adjustment*, and *Not usable* (see Fig 6). Maps where the algorithm was unable to produce a georeferenced result are considered as a sixth *Failed* category.

#### Evaluating georeferencing accuracy for real-world maps

In addition to testing our method on a wide range of realistic but simulated maps, we augment the simulated results with additional tests (333) for a sample of real-world maps. To do this we collected the top two maps listed on each of the country pages from the University of Texas at Austin Map Collection [96]—a total of 333 country-maps which contained toponyms. The sampled maps comprised a variety of image formats stored at low to medium levels of resolutions (the average width of the map images was approximately 1300 pixels). Since the true coordinates of these real world maps are unknown, we measured their accuracy as the *modelMax*_*LOO*_ leave-one-out maximum residual error (as contrasted to the true maximum error measured in our simulated cases).

## Results

### Simulated map georeferencing results

In total, the computing time required to process all 7,580 maps was approximately 61 cpu-hours or 2.5 days of consecutive computation, with a median of 28 seconds per map. A total of 6,430 maps were considered after excluding edge-case combinations of parameters unlikely to reflect real-world maps, such as cases with extreme toponym uncertainty (approaching 50% of the map extent). The results of our accuracy assessment are presented in Table 2 as the share of maps in each of the accuracy categories. To aide in the interpretation of the results we focus on the *trueMax* metric and the cumulative georeferencing success rates for two possible use-cases:

1. high-accuracy georeferencing as the share of maps in or better than the *Reasonable* accuracy category (less than 5% error); and
2. low-accuracy georeferencing as the share of maps in or better than the *Needs adjustment* category (less than 100% error).

**Table 2:** Accuracy result metrics from the automated georeferencing of simulated maps.

	*trueMax*		*modelMax*_*LOO*_		*modelMax*	
Accuracy	%	Cum.	%	Cum.	%	Cum.
**Full (n = 6,430):**						
Exc. (<1%)	12.6%	12.6%	33.0%	33.0%	51.0%	51.0%
Reas. (1–5%)	14.3%	26.9%	22.3%	55.4%	20.9%	71.9%
Approx. (5–20%)	24.6%	51.5%	21.5%	76.8%	15.8%	87.7%
Needs. (20–100%)	26.3%	77.7%	9.2%	86.1%	2.3%	90.0%
Not. (>100%)	12.2%	90.0%	3.9%	90.0%		
Failed	10.0%	100.0%	10.0%	100.0%	10.0%	100.0%
**Real. (n = 4,390):**						
Exc. (<1%)	17.6%	17.6%	45.9%	45.9%	60.0%	60.0%
Reas. (1–5%)	19.1%	36.7%	24.5%	70.4%	21.5%	81.5%
Approx. (5–20%)	25.6%	62.3%	16.5%	86.9%	12.1%	93.6%
Needs. (20–100%)	24.4%	86.7%	6.8%	93.7%	2.1%	95.7%
Not. (>100%)	9.0%	95.7%	2.1%	95.7%		
Failed	4.3%	100.0%	4.3%	100.0%	4.3%	100.0%
**Hi-res. (n = 3,512):**						
Exc. (<1%)	19.8%	19.8%	49.5%	49.5%	60.8%	60.8%
Reas. (1–5%)	20.2%	40.1%	26.1%	75.7%	22.9%	83.7%
Approx. (5–20%)	26.9%	67.0%	16.1%	91.8%	12.3%	96.1%
Needs. (20–100%)	24.5%	91.4%	5.3%	97.1%	2.1%	98.2%
Not. (>100%)	6.8%	98.2%	1.1%	98.2%		
Failed	1.8%	100.0%	1.8%	100.0%	1.8%	100.0%

Shows percent and cumulative percent of simulated maps in each accuracy category, for three subsets of maps: the “full” sample; a “realistic” sample with at least 20 toponyms and toponym uncertainty no larger than 10km; and a “high resolution” group of realistic maps that also have a pixel resolution of 2000 or higher.

In terms of overall success rates, approximately one-fourth (26.9%) of the 6,430 simulated maps could be used for high-accuracy georeferencing (less than 5% error), while about three-fourths of maps (77.7%) could be used for low-accuracy georeferencing (less than 100% error). However, the full sample of simulated maps includes maps unlikely to be encountered in the real world as well as maps of poor quality. Therefore, we additionally report what levels of accuracy to expect for a subset of more *realistic* maps—dropping outlier combinations of bad image quality and low resolution—as well as a subset of *high-resolution* realistic maps (see Table 2 for details on the definitions of each subset of maps). For the more realistic map sample (N = 4,390), over one-third (36.7%) of maps can be georeferenced with high accuracy, and 86.7% with low accuracy. For the high-resolution, realistic map sample (N = 3,512), 40.1% are georeferenced with high accuracy, and nine out of ten maps (91.4%) with low accuracy. The automated model selection procedure tended to favor lower-order polynomial models, with 1st order polynomials used in 69% of all georeferenced maps, followed by 24% of maps for 2nd order polynomials, and only 7% for 3rd order polynomials.

**Fig 7:**
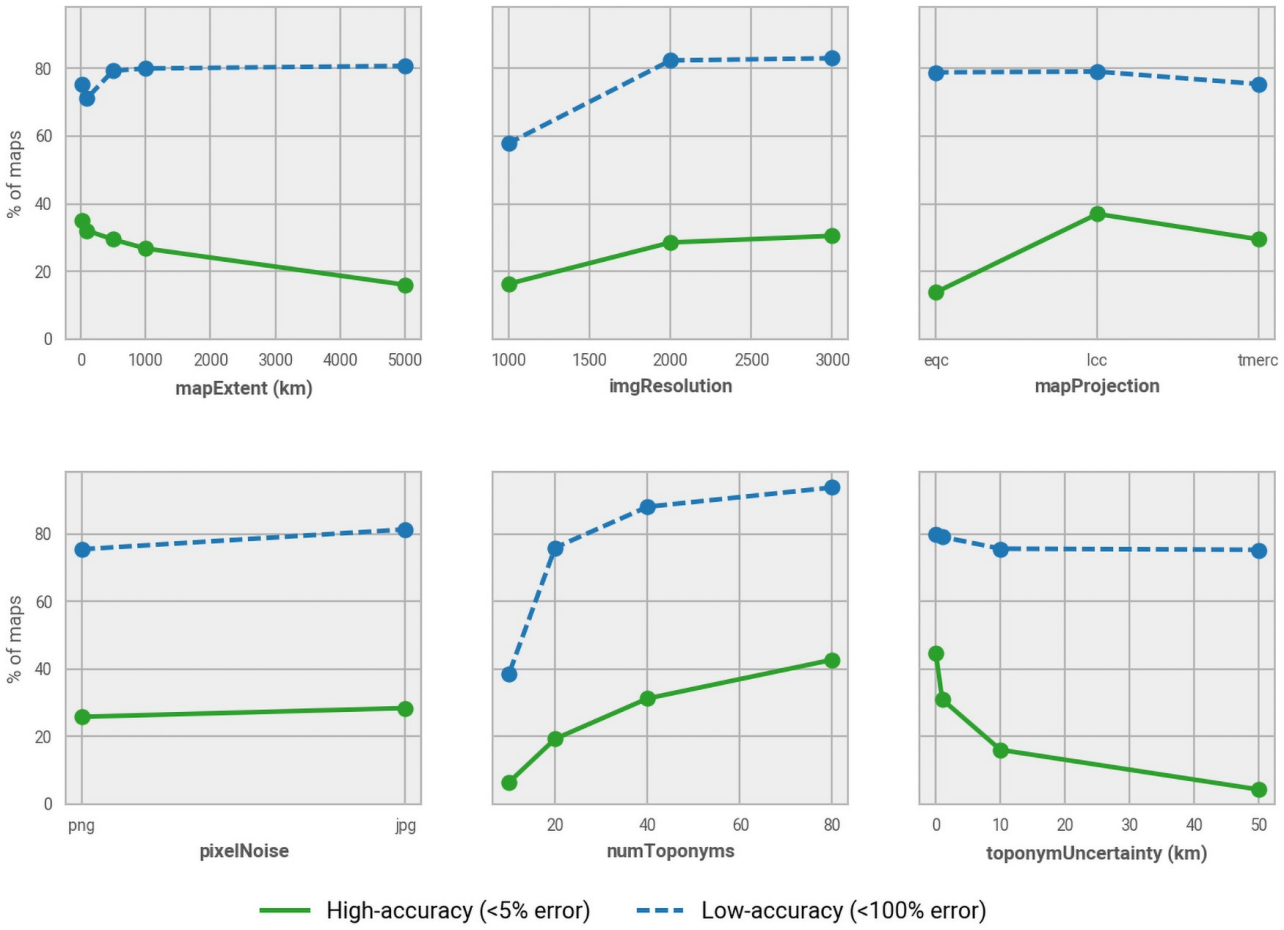
Effect of simulated map parameters for georeferencing success rates. Share of all simulated maps that were successfully georeferenced, for different parameter values.

Going beyond these topline values, we can use the simulation parameters we defined for each map to evaluate the characteristics of maps that most negatively affect the accuracy of toponym-based map georeferencing (see Fig 7). For low-accuracy georeferencing only two of the map parameters have a noticeable impact. The first and most important factor is that fewer numbers of toponyms is related to lower georeferencing success rates, particularly for maps with only 10 toponyms (with accuracy dropping from about 80% in cases with a high number of toponyms, to 40% with smaller numbers of toponyms). Second, we see that very coarse image resolutions result in a drop of georeferencing success from 80% to 60%. Only when high-accuracy georeferencing is required do we see that toponym uncertainty, map extent, or map projection have an effect on the success rates.

Recognizing that our metric of accuracy can only be used in a simulation setting, Table 2 further presents the results for two alternative metrics of accuracy in which only control points are used to assess accuracy, to allow for meaningful comparison to real-world cases. In terms of the share of simulated maps that can be georeferenced to within 1% error, the traditionally applied model residual calculation (*modelMax*) suggests a success rate of 51%, while the calculation based on leave-one-out residuals (*modelMax*_*LOO*_) suggests a success rate of 33%. Although both of these metrics are overly optimistic—i.e., they contrast to the known success rate (*trueMax*) of 12.6%—they may provide guidance to users seeking to compare the results from this paper to georeferencing results from the real world where the *trueMax* georeferencing error is unknown.

### Real world georeferencing results

**Fig 8:**
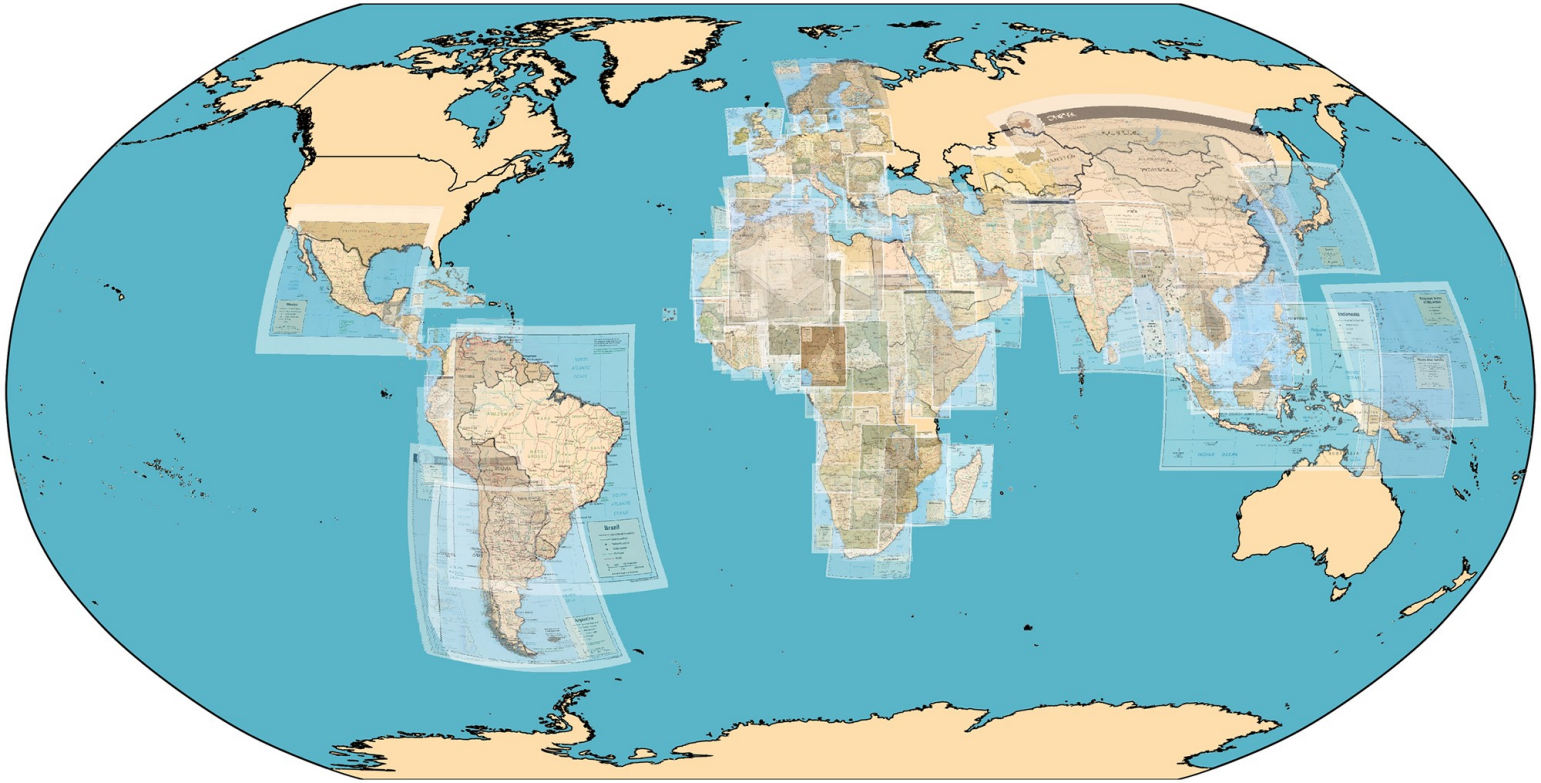
Georeferencing results for real-world country maps. Shows subset of maps with errors less than 5% of map radius, representing about 67% of the total sample. The georeferenced overlay maps are from the University of Texas at Austin’s Perry-Castañeda Library (PCL) Map Collection and are in the public domain. The complete list of all map images and their source URLs can be found in the replication data accompanying this article (see Data Availability statement). The geodata used to render country outlines is from ©Natural Earth data and is in the public domain.

In addition to our simulation tests, we also tested the capabilities of toponym based georeferencing for a set of real-world maps (Table 3). The country coverage and overall accuracy of the resulting georeferenced outputs are visualized in Fig 8. A representative sample of the results for individual country maps are included as figures in the Supporting information section.

**Table 3:** Accuracy result metrics from the automated georeferencing of real-world country-maps.

	*modelMax*_*LOO*_		*modelMax*	
Accuracy	%	Cum.	%	Cum.
Exc. (<1%)	44.4%	44.4%	63.1%	63.1%
Reas. (1–5%)	22.8%	67.3%	15.3%	78.4%
Approx. (5–20%)	7.2%	74.5%	3.9%	82.3%
Needs. (20–100%)	7.8%	82.3%	2.4%	84.7%
Not. (>100%)	2.4%	84.7%	0.0%	100%
Failed	15.3%	100.0%	15.3%	100.0%

Shows percent and cumulative percent of real-world maps (n = 333) in each accuracy category.

Based on the *modelMax*_*LOO*_ error metric, out of a total of 333 maps, approximately 82% of the maps resulted in low-accuracy georeferencing outputs (less than 100% error); 67% of the maps resulted in high-accuracy georeferenced outputs (less than 5% error); and 44% of the total had less than 1% error. This is nearly identical to the *modelMax*_*LOO*_ results for the simulated dataset (see Table 2).

## Discussion

### Capabilities & limitations

Our results illustrate a number of advantages of—and limitations to—toponym-based approaches to georeferencing. Broadly, we find that toponym-based georeferencing can be used to georeference most contemporary and recent historical maps, including both overview maps and topographic map sheets that contain toponyms. Maps containing text of any language and script can be read using this method, provided they use clear text label typesetting. As few as 10 toponyms was shown to be sufficient in order to achieve a generally acceptable level of georeferencing accuracy.

Toponym-based georeferencing is—by design—sensitive to image resolution, but more effective at lower resolutions than anticipated. This is predominantly because the primary need of the algorithm is the ability to discern text, which our findings suggest does not degrade until text resolutions become lower than 1.33 pixels-per-point of font size (the international standard). This is reflected in our results, in which an image resolution of 1000 pixels was equivalent to approximately 1 pixel-per-point (or 75% of the standard size); in these cases, only 60% of maps were succesfully georeferenced, down from over 80% in cases where text resolution was at least 1.33 pixels-per-point (our 2000 and 3000 pixel resolution cases).

A second area of anticipated sensitivity was related to toponym uncertainty—i.e., disagreement across gazetteer sources as to where places are located. As the spatial extent of the map to be georeferenced decreased, we anticipated these disagreements would result in larger potential inaccuracy. Our results, however, suggest that toponym-assisted georeferencing can produce accurate results for map extents as small as 25km, and simulated toponym uncertainties of up to 10km. This apparent strength of the model is due to both (a) the reliance on a pattern matching strategy, in which multiple places would need to have bias in similar geographic directions, and (b) a generally small level of disagreement across gazetteers, frequently within 1km [97, 98]. Despite this promise, based on anecdotal testing we caution against using the approach presented in this paper for maps smaller than 25km due to the sparsity of meaningful toponyms likely to be available at these scales.

Although distortions from map projections may negatively impact the toponym matching process in some cases, this should not be a concern for most maps. Our results showed no noticeable difference between several widely used map projections for low-accuracy georeferencing (less than 100% error of map radius), and only minor differences for high-accuracy georeferencing (less than 5% error). The effects of map projection also appear to be limited to high-accuracy georeferencing of very large extent maps, e.g. global or regional maps where distortions due to map projection should be the most pronounced [99]. However, our testing in this dimension has been limited to map projections that are commonly encountered, and we would caution against generalizing our results to less common source projections.

### Future work

There are several elements of the presented methodology that could be improved as next steps for future research. These include improvements to: a) the detection and extraction of toponym text labels and their image locations, b) approaches for geocoding and matching toponyms to candidate real-world coordinates, and c) methods for refining the lists of control points and selecting between multiple possible transform functions.

Recognizing that the number of toponyms identified within the map was a key predictor of georeferencing accuracy, we suggest it should be a focus of researchers interested in improving this approach. Text-recognition in maps is a field which is rapidly evolving [5], with several alternative approaches to the implementation presented here that could help improve the georeferencing results. The approach to text recognition described in this paper could be compared with the use of existing tools and software for map-based text recognition [67], machine-learning approaches designed for complex multi-color text detection [62], and predictive toponym detection given the approximate coordinates from an initial round of georeferencing [55, 56, 63, 100]. Following text-recognition, more sophisticated symbol recognition of toponym markers in the image [76], as well as improved linking of toponym labels with toponym markers [101], would likely result in more accurately detected toponym locations and higher overall accuracy results.

Even with sufficient numbers of toponyms, the approaches used in this method for matching and transform estimation still resulted in some cases of falsely matched toponyms and distorted transformations. Possible avenues for improved matching include repeated step-wise georeferencing to incrementally refine and limit the candidates to be searched [55, 56, 58] or the use of more sophisticated point set registration methods [56, 87–89]. Assuming a set of matched points, there are also a number of alternative non-polynomial transform estimation methods that have been suggested [99, 102, 103] that may or may not result in more accurate map transformations, particularly for larger-extent maps.

## Conclusion

Georeferencing mapped documents is an important step in the process of making archival and contemporaneous maps discoverable, searchable, and otherwise accessible [30, 35, 42, 104]. However, the process of georeferencing is—even today—largely manual and inefficient.

Building on past literature, this study sought to answer the question: *to what degree can map georeferencing be automated through the use of map toponym labels*? In answering this question, we made three contributions to the literature. First, we outlined a new, automated approach to georeferencing that reads and parses the names of toponyms listed on a map, searches and retrieves the real-world coordinates of these places, and uses this information to estimate the coordinate reference frame of the map. Second, by evaluating this approach on a large sample of simulated and real-world maps, we demonstrated that the method is sufficiently accurate to be used for real-world and general-purpose use-cases. Third, we made the methodology readily available as open-source code for researchers who wish to replicate or improve the technique (see Data Availability statement), and as a web-based tool for end-users who wish to use it to georeference maps.

The methodology demonstrated here was—with no manual intervention or tuning—able to automatically process and provide results nearly indistinguishable from manually georeferenced maps (accurate to within 5% of the map radius) in 40% of cases. In 90% of cases, the toponym-based georeferencing approach was able to provide maps referenced to broadly correct regions of the world, with errors that—while substantial—may be easily correctable with small perturbations by human coders. These results were robust against a variety of simulated and real-world map parameters, able to georeference low-information maps with as few as 10 toponym labels, image resolutions as low as 1000 pixels, map extents as small as 25 km across, and several commonly available map projections.

As institutions continue to create, find, archive, and digitize mapped documents, the need for automated procedures to georeference that information will continue to grow. The work presented in this paper illustrates that toponym-based approaches to georeferencing may provide an automated solution to this challenge, and one which is applicable to a broad range of cartographic presentations of information. Replication code provided with this study is open-source and can be used freely by researchers, libraries, and museums for automated toponym-assisted georeferencing of large collections of maps.

## Supporting information

S1 Fig Benin country map. Automatically georeferenced map and control points overlaid on satellite imagery. Map resolution = 1046 x 1227 pixels. *ModelMax*_*LOO*_ = 0.8 pixels (0.1% of image radius). The map image is from the University of Texas at Austin’s Perry-Castañeda Library (PCL) Map Collection and is in the public domain: https://legacy.lib.utexas.edu/maps/africa/benin_pol_2007.jpg. The background satellite data is from NASA Visible Earth’s “Blue Marble” true-color global image mosaic and is in the public domain. The geodata used to render country outlines (in white) and roads (in yellow) is from ©Natural Earth data and is in the public domain.
(PNG)

S2 Fig Cape Verde country map. Automatically georeferenced map and control points overlaid on satellite imagery. Map resolution = 2584 x 2003 pixels. *ModelMax*_*LOO*_ = 2.3 pixels (0.14% of image radius). The map image is from the University of Texas at Austin’s Perry-Castañeda Library (PCL) Map Collection and is in the public domain: http://legacy.lib.utexas.edu/maps/africa/cape_verde_physio-2004.jpg. The background satellite data is from NASA Visible Earth’s “Blue Marble” true-color global image mosaic and is in the public domain. The geodata used to render country outlines (in white) and roads (in yellow) is from ©Natural Earth data and is in the public domain.
(PNG)

S3 Fig Iran country map. Automatically georeferenced map and control points overlaid on satellite imagery. Map resolution = 2000 x 2001 pixels. *ModelMax*_*LOO*_ = 2.1 pixels (0.15% of image radius). The map image is from the University of Texas at Austin’s Perry-Castañeda Library (PCL) Map Collection and is in the public domain: http://legacy.lib.utexas.edu/maps/middle_east_and_asia/iran_physio-2001.jpg. The background satellite data is from NASA Visible Earth’s “Blue Marble” true-color global image mosaic and is in the public domain. The geodata used to render country outlines (in white) and roads (in yellow) is from ©Natural Earth data and is in the public domain.
(PNG)

S4 Fig Liberia country map. Automatically georeferenced map and control points overlaid on satellite imagery. Map resolution = 1984 x 2452 pixels. *ModelMax*_*LOO*_ = 2.4 pixels (0.15% of image radius). The map image is from the University of Texas at Austin’s Perry-Castañeda Library (PCL) Map Collection and is in the public domain: http://legacy.lib.utexas.edu/maps/africa/liberia_physio-2004.jpg. The background satellite data is from NASA Visible Earth’s “Blue Marble” true-color global image mosaic and is in the public domain. The geodata used to render country outlines (in white) and roads (in yellow) is from ©Natural Earth data and is in the public domain.
(PNG)

S5 Fig Indonesia country map. Automatically georeferenced map and control points overlaid on satellite imagery. Map resolution = 1389 x 939 pixels. *ModelMax*_*LOO*_ = 1.4 pixels (0.16% of image radius). The map image is from the University of Texas at Austin’s Perry-Castañeda Library (PCL) Map Collection and is in the public domain: https://legacy.lib.utexas.edu/maps/middle_east_and_asia/indonesia_pol_2002.jpg. The background satellite data is from NASA Visible Earth’s “Blue Marble” true-color global image mosaic and is in the public domain. The geodata used to render country outlines (in white) and roads (in yellow) is from ©Natural Earth data and is in the public domain.
(PNG)

S6 Fig Denmark country map. Automatically georeferenced map and control points overlaid on satellite imagery. Map resolution = 1275 x 1036 pixels. *ModelMax*_*LOO*_ = 8.3 pixels (1.0% of image radius). The map image is from the University of Texas at Austin’s Perry-Castañeda Library (PCL) Map Collection and is in the public domain: https://legacy.lib.utexas.edu/maps/europe/denmark_pol81.jpg. The background satellite data is from NASA Visible Earth’s “Blue Marble” true-color global image mosaic and is in the public domain. The geodata used to render country outlines (in white) and roads (in yellow) is from ©Natural Earth data and is in the public domain.
(PNG)

S7 Fig Equitorial Guinea country map. Automatically georeferenced map and control points overlaid on satellite imagery. Map resolution = 1355 x 1657 pixels. *ModelMax*_*LOO*_ = 10.9 pixels (1.0% of image radius). The map image is from the University of Texas at Austin’s Perry-Castañeda Library (PCL) Map Collection and is in the public domain: https://legacy.lib.utexas.edu/maps/africa/equatorial_guinea_pol_1992.jpg. The background satellite data is from NASA Visible Earth’s “Blue Marble” true-color global image mosaic and is in the public domain. The geodata used to render country outlines (in white) and roads (in yellow) is from ©Natural Earth data and is in the public domain.
(PNG)

S8 Fig Portugal country map. Automatically georeferenced map and control points overlaid on satellite imagery. Map resolution = 1042 x 1318 pixels. *ModelMax*_*LOO*_ = 8.8 pixels (1.04% of image radius). The map image is from the University of Texas at Austin’s Perry-Castañeda Library (PCL) Map Collection and is in the public domain: https://legacy.lib.utexas.edu/maps/europe/portugal.jpg. The background satellite data is from NASA Visible Earth’s “Blue Marble” true-color global image mosaic and is in the public domain. The geodata used to render country outlines (in white) and roads (in yellow) is from ©Natural Earth data and is in the public domain.
(PNG)

S9 Fig Ecuador country map. Automatically georeferenced map and control points overlaid on satellite imagery. Map resolution = 2023 x 2692 pixels. *ModelMax*_*LOO*_ = 17.8 pixels (1.05% of image radius). The map image is from the University of Texas at Austin’s Perry-Castañeda Library (PCL) Map Collection and is in the public domain: https://legacy.lib.utexas.edu/maps/americas/txu-pclmaps-oclc-785902207-ecuador_pol-2011.jpg. The background satellite data is from NASA Visible Earth’s “Blue Marble” true-color global image mosaic and is in the public domain. The geodata used to render country outlines (in white) and roads (in yellow) is from ©Natural Earth data and is in the public domain.
(PNG)

S10 Fig Mexico country map. Automatically georeferenced map and control points overlaid on satellite imagery. Map resolution = 1248 x 1010 pixels. *ModelMax*_*LOO*_ = 64.3 pixels (8.0% of image radius). The map image is from the University of Texas at Austin’s Perry-Castañeda Library (PCL) Map Collection and is in the public domain: https://legacy.lib.utexas.edu/maps/americas/mexico.gif. The background satellite data is from NASA Visible Earth’s “Blue Marble” true-color global image mosaic and is in the public domain. The geodata used to render country outlines (in white) and roads (in yellow) is from ©Natural Earth data and is in the public domain.
(PNG)
