# Supplementary figures and images for: Toponym-assisted map georeferencing: Evaluating the use of toponyms for the digitization of map collections
Source: PLoS One. 2021 Nov 18;16(11):e0260039. doi: 10.1371/journal.pone.0260039 (PMC8601528; doi:10.1371/journal.pone.0260039)

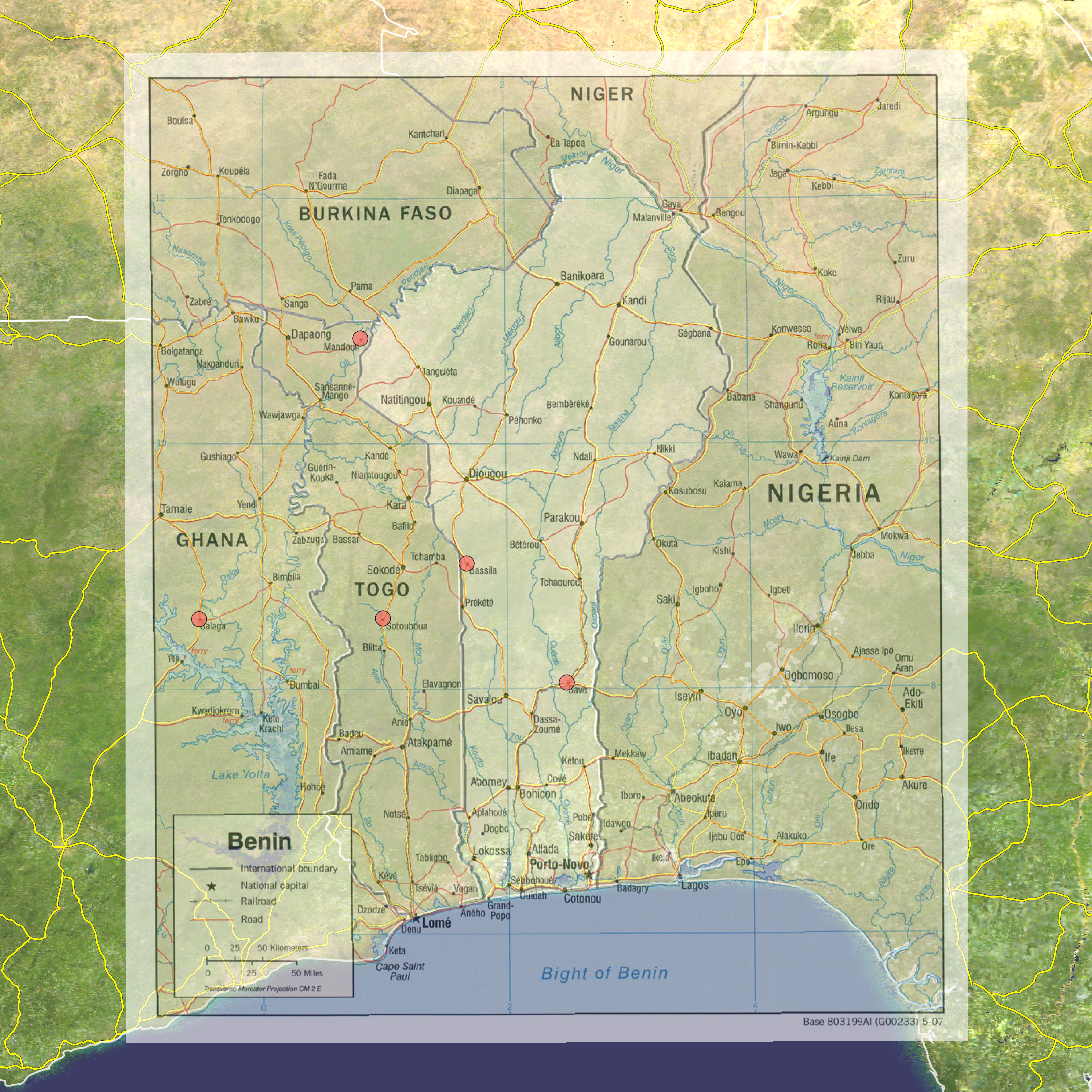

Supplement: S1 Fig — Automatically georeferenced map and control points overlaid on satellite imagery. Map resolution = 1046 x 1227 pixels. ModelMaxLOO = 0.8 pixels (0.1% of image radius). The map image is from the University of Texas at Austin’s Perry-Castañeda Library (PCL) Map Collection and is in the public domain: https://legacy.lib.utexas.edu/maps/africa/benin_pol_2007.jpg. The background satellite data is from NASA Visible Earth’s “Blue Marble” true-color global image mosaic and is in the public domain. The geodata used to render country outlines (in white) and roads (in yellow) is from ©Natural Earth data and is in the public domain. (PNG) [file pone.0260039.s001.png]

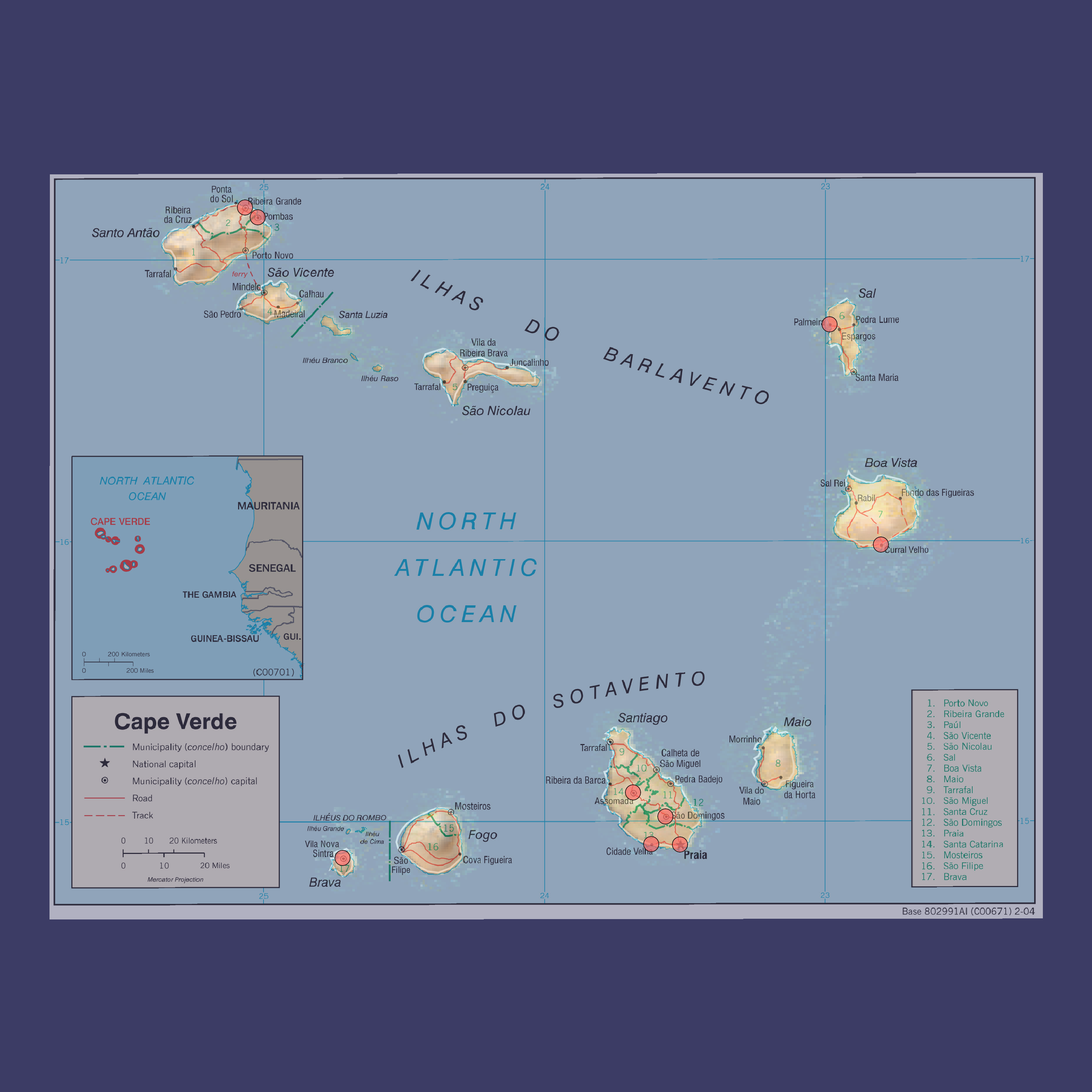

Supplement: S2 Fig — Automatically georeferenced map and control points overlaid on satellite imagery. Map resolution = 2584 x 2003 pixels. ModelMaxLOO = 2.3 pixels (0.14% of image radius). The map image is from the University of Texas at Austin’s Perry-Castañeda Library (PCL) Map Collection and is in the public domain: http://legacy.lib.utexas.edu/maps/africa/cape_verde_physio-2004.jpg. The background satellite data is from NASA Visible Earth’s “Blue Marble” true-color global image mosaic and is in the public domain. The geodata used to render country outlines (in white) and roads (in yellow) is from ©Natural Earth data and is in the public domain. (PNG) [file pone.0260039.s002.png]

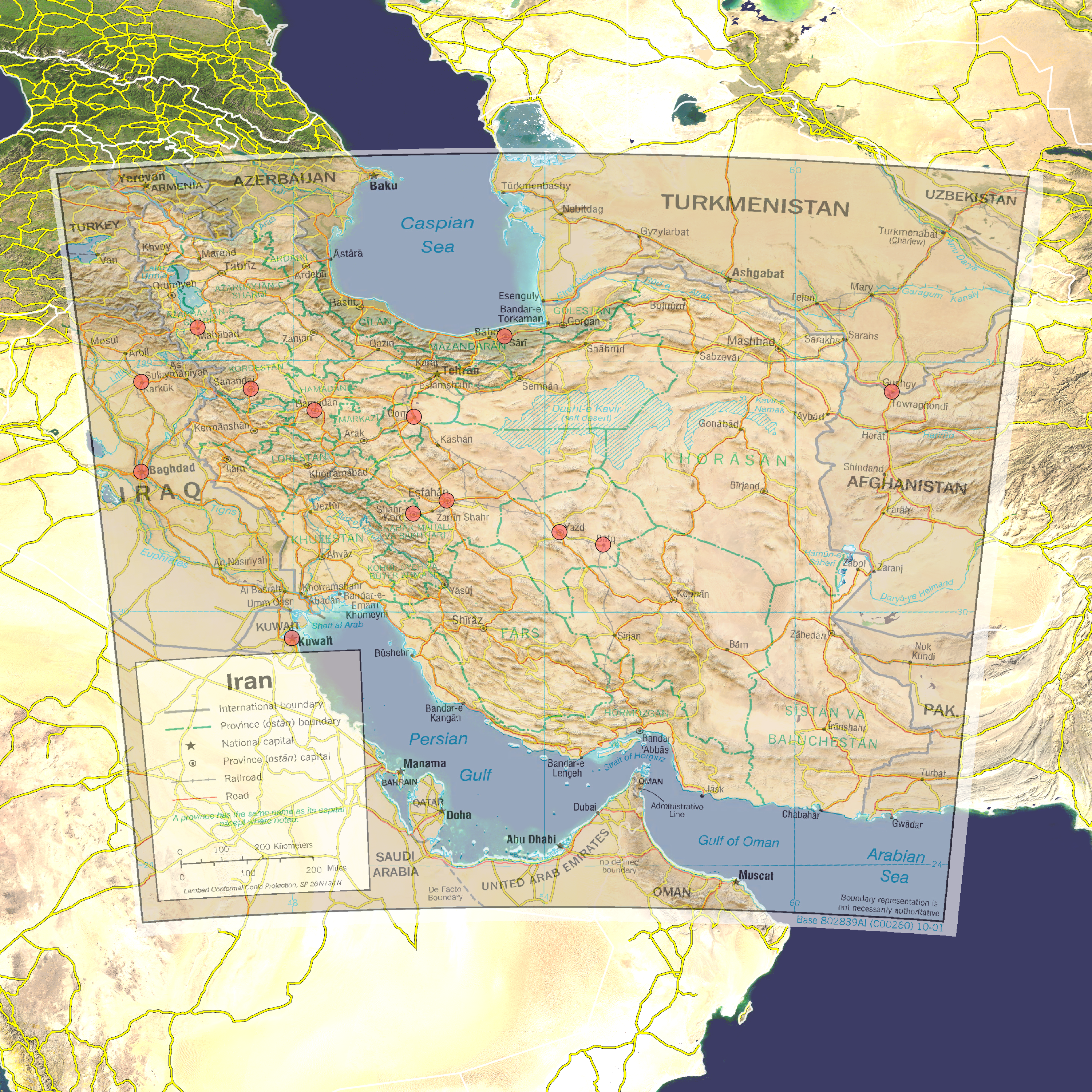

Supplement: S3 Fig — Automatically georeferenced map and control points overlaid on satellite imagery. Map resolution = 2000 x 2001 pixels. ModelMaxLOO = 2.1 pixels (0.15% of image radius). The map image is from the University of Texas at Austin’s Perry-Castañeda Library (PCL) Map Collection and is in the public domain: http://legacy.lib.utexas.edu/maps/middle_east_and_asia/iran_physio-2001.jpg. The background satellite data is from NASA Visible Earth’s “Blue Marble” true-color global image mosaic and is in the public domain. The geodata used to render country outlines (in white) and roads (in yellow) is from ©Natural Earth data and is in the public domain. (PNG) [file pone.0260039.s003.png]

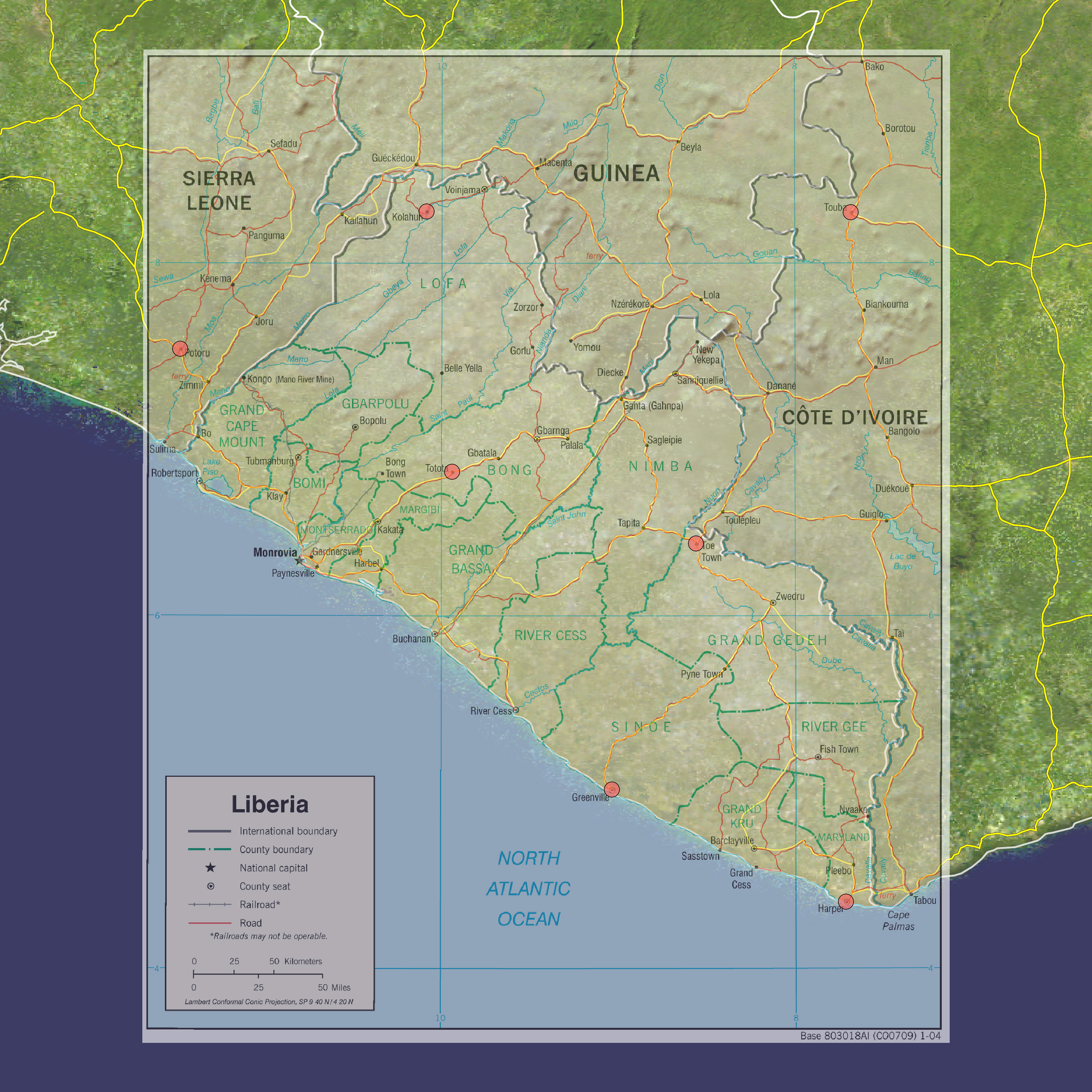

Supplement: S4 Fig — Automatically georeferenced map and control points overlaid on satellite imagery. Map resolution = 1984 x 2452 pixels. ModelMaxLOO = 2.4 pixels (0.15% of image radius). The map image is from the University of Texas at Austin’s Perry-Castañeda Library (PCL) Map Collection and is in the public domain: http://legacy.lib.utexas.edu/maps/africa/liberia_physio-2004.jpg. The background satellite data is from NASA Visible Earth’s “Blue Marble” true-color global image mosaic and is in the public domain. The geodata used to render country outlines (in white) and roads (in yellow) is from ©Natural Earth data and is in the public domain. (PNG) [file pone.0260039.s004.png]

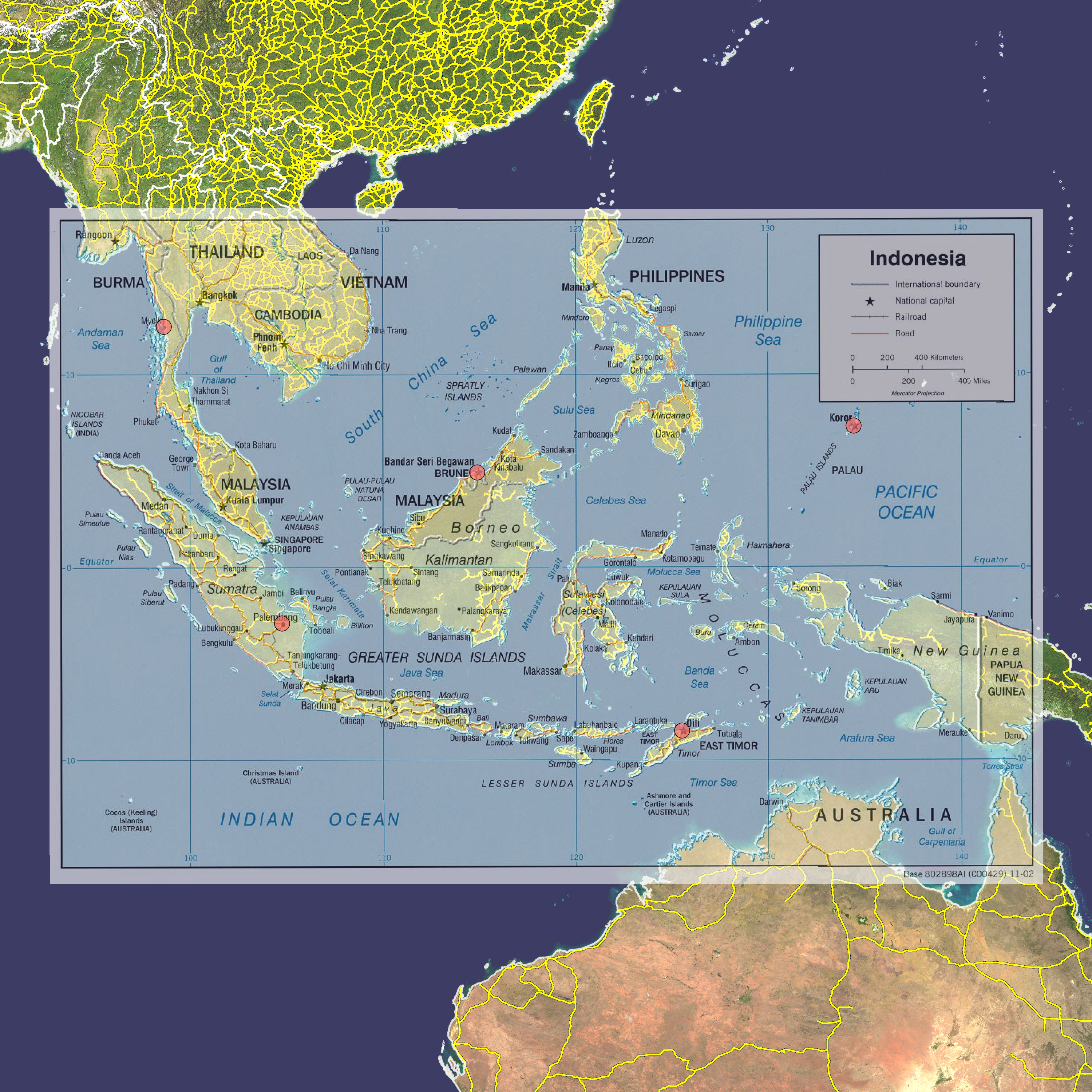

Supplement: S5 Fig — Automatically georeferenced map and control points overlaid on satellite imagery. Map resolution = 1389 x 939 pixels. ModelMaxLOO = 1.4 pixels (0.16% of image radius). The map image is from the University of Texas at Austin’s Perry-Castañeda Library (PCL) Map Collection and is in the public domain: https://legacy.lib.utexas.edu/maps/middle_east_and_asia/indonesia_pol_2002.jpg. The background satellite data is from NASA Visible Earth’s “Blue Marble” true-color global image mosaic and is in the public domain. The geodata used to render country outlines (in white) and roads (in yellow) is from ©Natural Earth data and is in the public domain. (PNG) [file pone.0260039.s005.png]

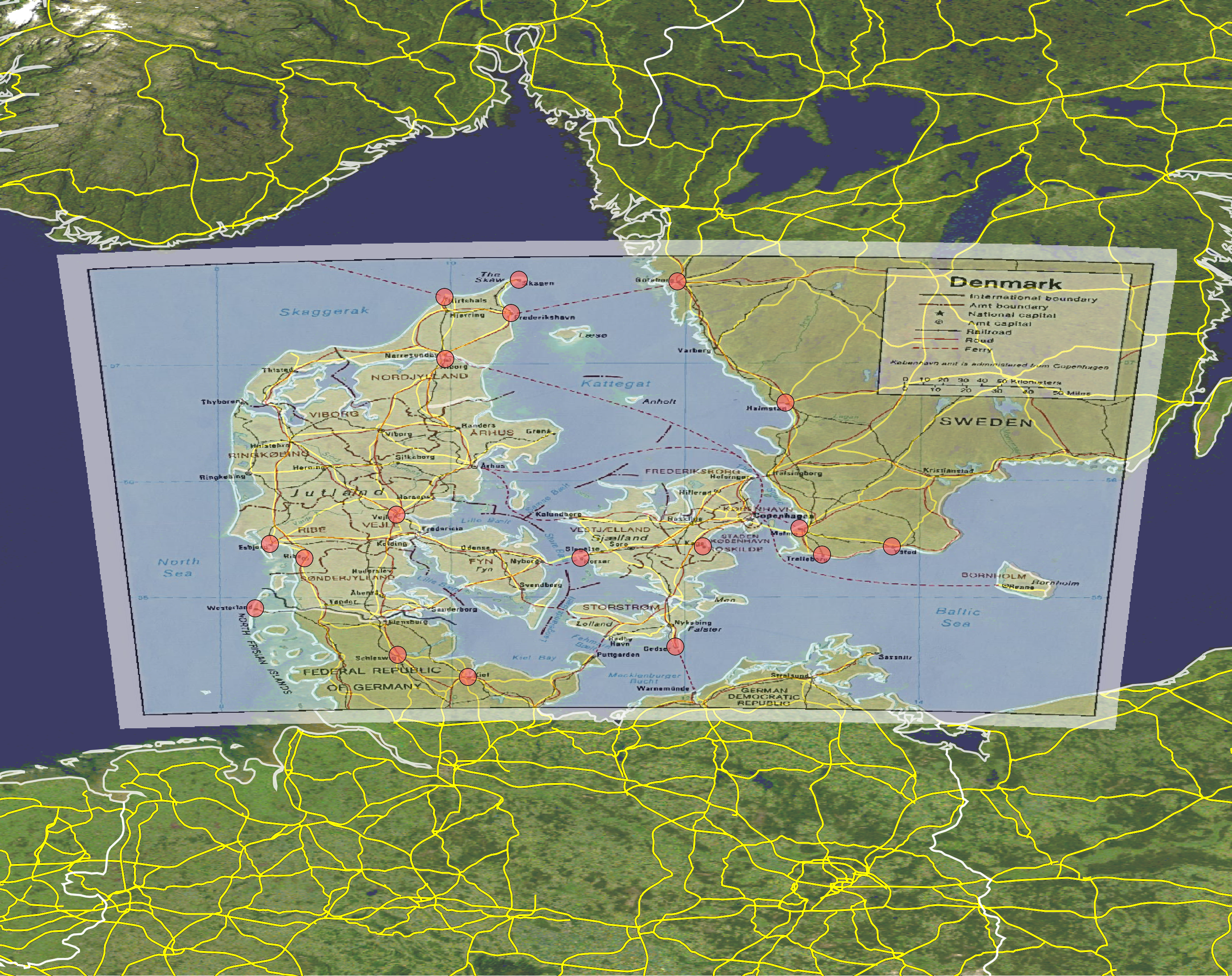

Supplement: S6 Fig — Automatically georeferenced map and control points overlaid on satellite imagery. Map resolution = 1275 x 1036 pixels. ModelMaxLOO = 8.3 pixels (1.0% of image radius). The map image is from the University of Texas at Austin’s Perry-Castañeda Library (PCL) Map Collection and is in the public domain: https://legacy.lib.utexas.edu/maps/europe/denmark_pol81.jpg. The background satellite data is from NASA Visible Earth’s “Blue Marble” true-color global image mosaic and is in the public domain. The geodata used to render country outlines (in white) and roads (in yellow) is from ©Natural Earth data and is in the public domain. (PNG) [file pone.0260039.s006.png]

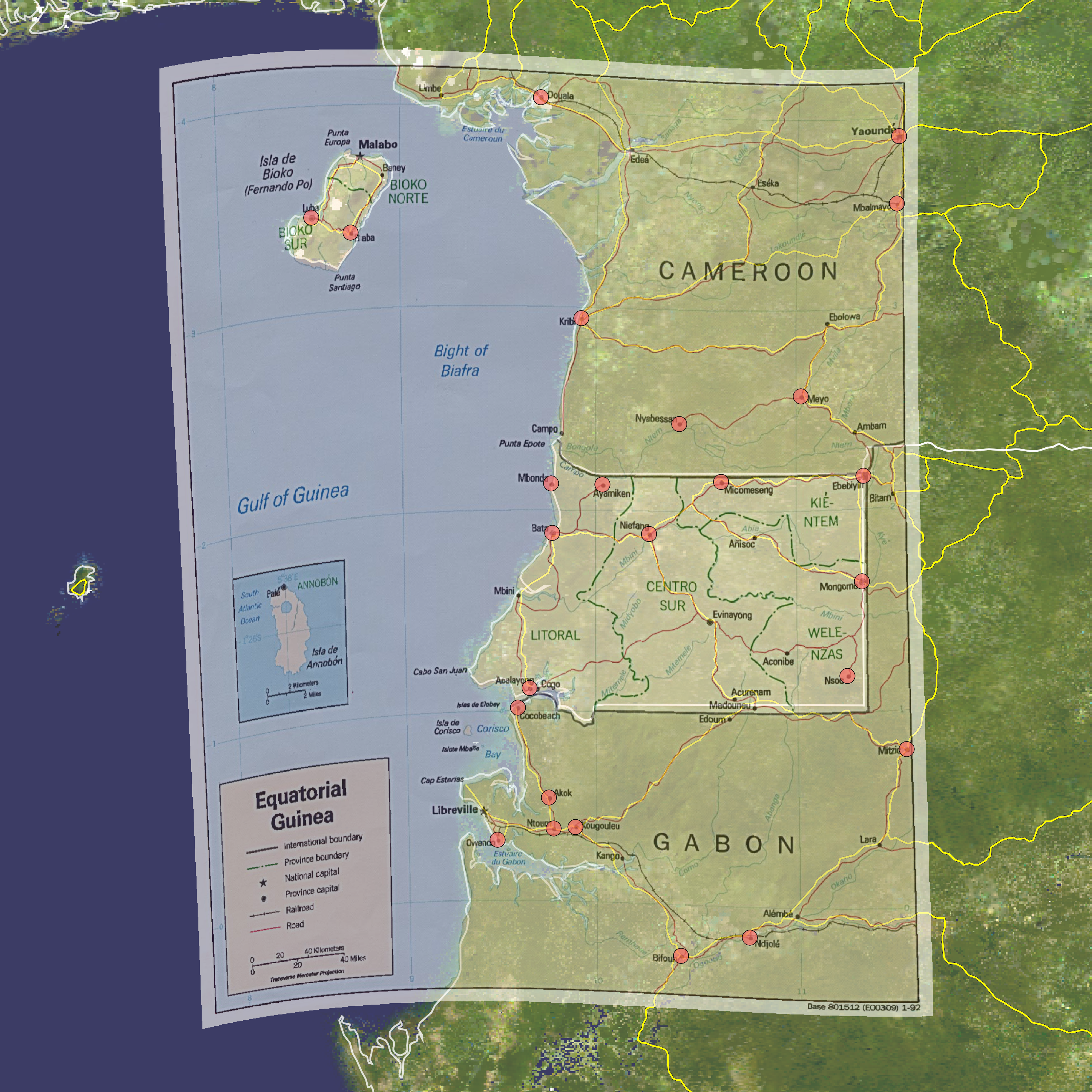

Supplement: S7 Fig — Automatically georeferenced map and control points overlaid on satellite imagery. Map resolution = 1355 x 1657 pixels. ModelMaxLOO = 10.9 pixels (1.0% of image radius). The map image is from the University of Texas at Austin’s Perry-Castañeda Library (PCL) Map Collection and is in the public domain: https://legacy.lib.utexas.edu/maps/africa/equatorial_guinea_pol_1992.jpg. The background satellite data is from NASA Visible Earth’s “Blue Marble” true-color global image mosaic and is in the public domain. The geodata used to render country outlines (in white) and roads (in yellow) is from ©Natural Earth data and is in the public domain. (PNG) [file pone.0260039.s007.png]

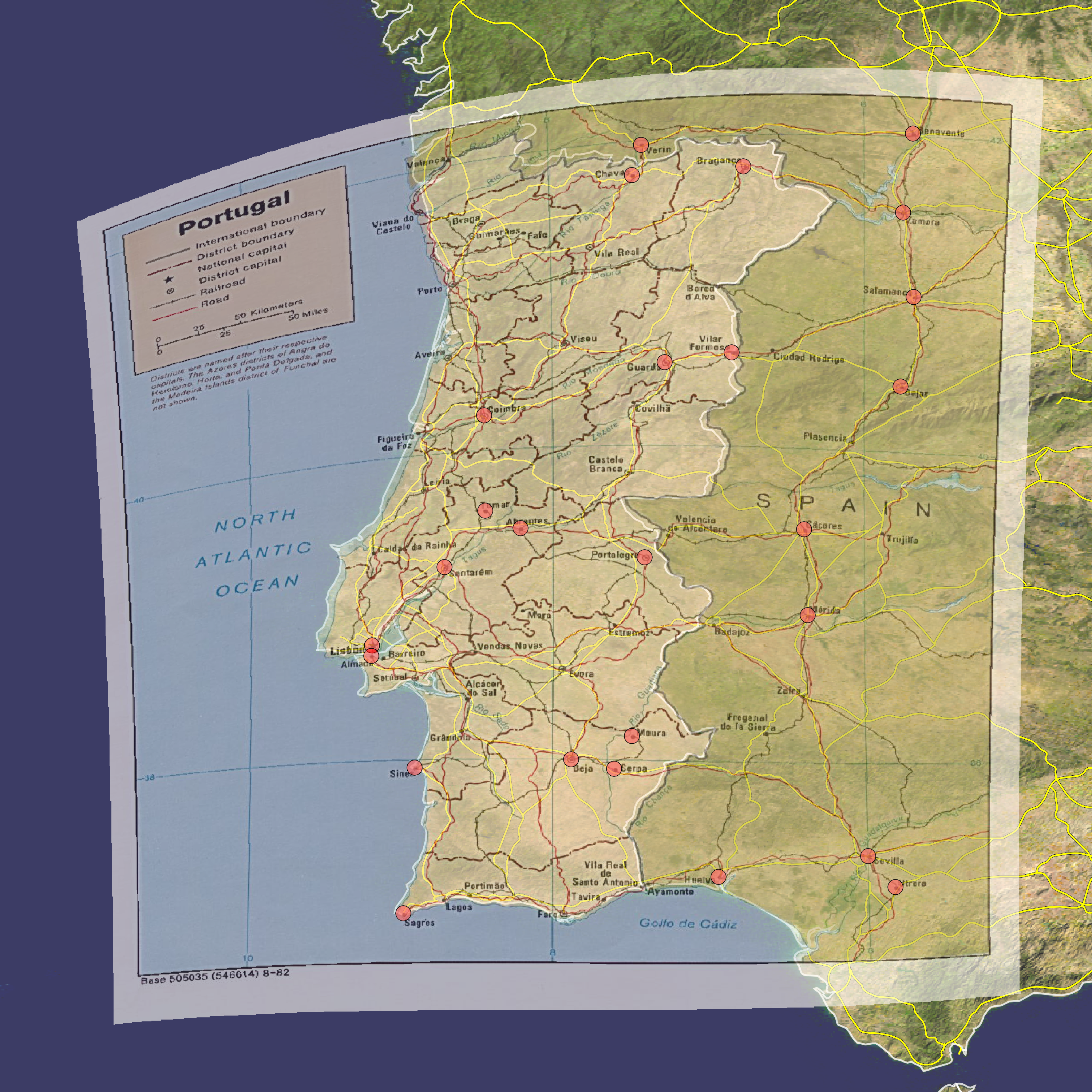

Supplement: S8 Fig — Automatically georeferenced map and control points overlaid on satellite imagery. Map resolution = 1042 x 1318 pixels. ModelMaxLOO = 8.8 pixels (1.04% of image radius). The map image is from the University of Texas at Austin’s Perry-Castañeda Library (PCL) Map Collection and is in the public domain: https://legacy.lib.utexas.edu/maps/europe/portugal.jpg. The background satellite data is from NASA Visible Earth’s “Blue Marble” true-color global image mosaic and is in the public domain. The geodata used to render country outlines (in white) and roads (in yellow) is from ©Natural Earth data and is in the public domain. (PNG) [file pone.0260039.s008.png]

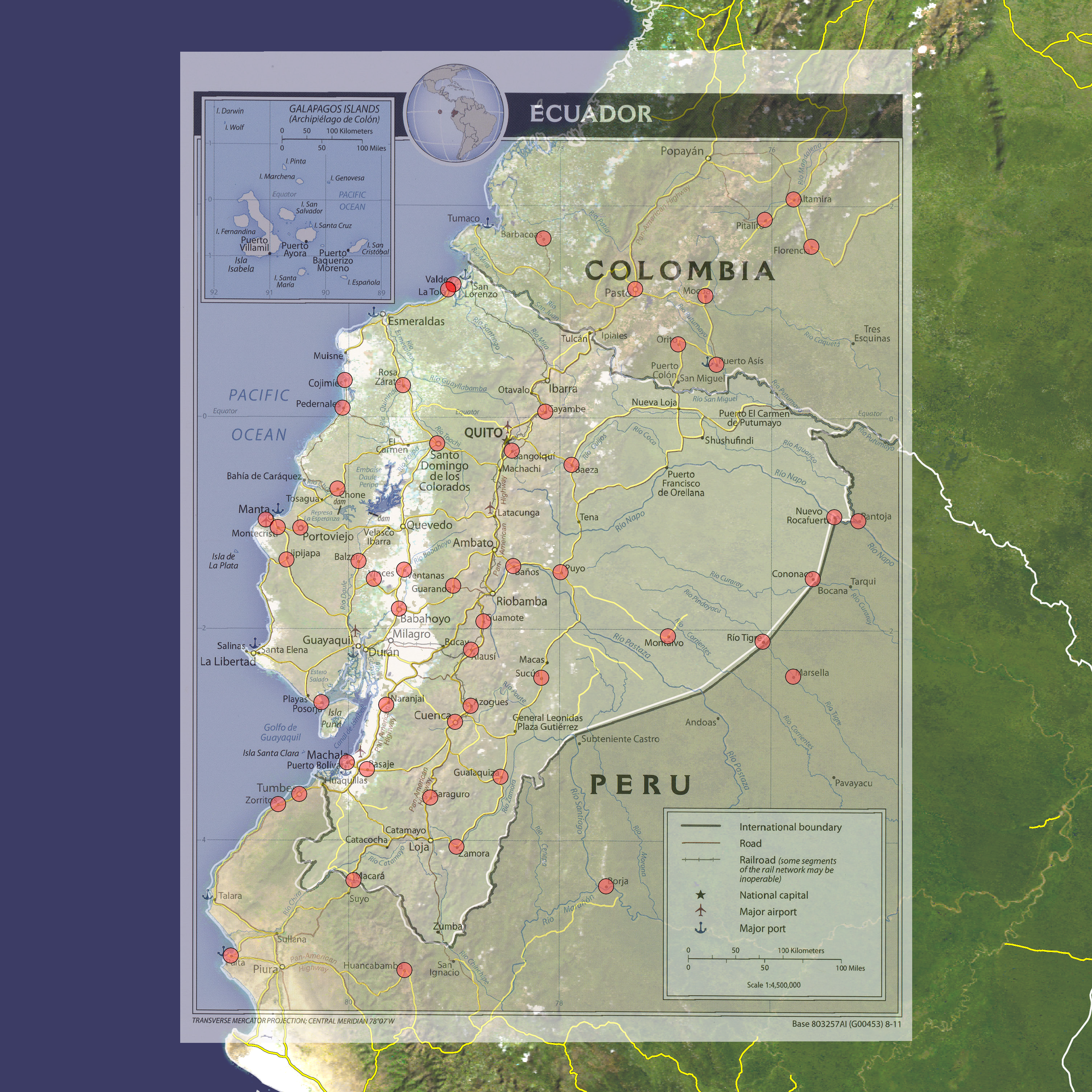

Supplement: S9 Fig — Automatically georeferenced map and control points overlaid on satellite imagery. Map resolution = 2023 x 2692 pixels. ModelMaxLOO = 17.8 pixels (1.05% of image radius). The map image is from the University of Texas at Austin’s Perry-Castañeda Library (PCL) Map Collection and is in the public domain: https://legacy.lib.utexas.edu/maps/americas/txu-pclmaps-oclc-785902207-ecuador_pol-2011.jpg. The background satellite data is from NASA Visible Earth’s “Blue Marble” true-color global image mosaic and is in the public domain. The geodata used to render country outlines (in white) and roads (in yellow) is from ©Natural Earth data and is in the public domain. (PNG) [file pone.0260039.s009.png]

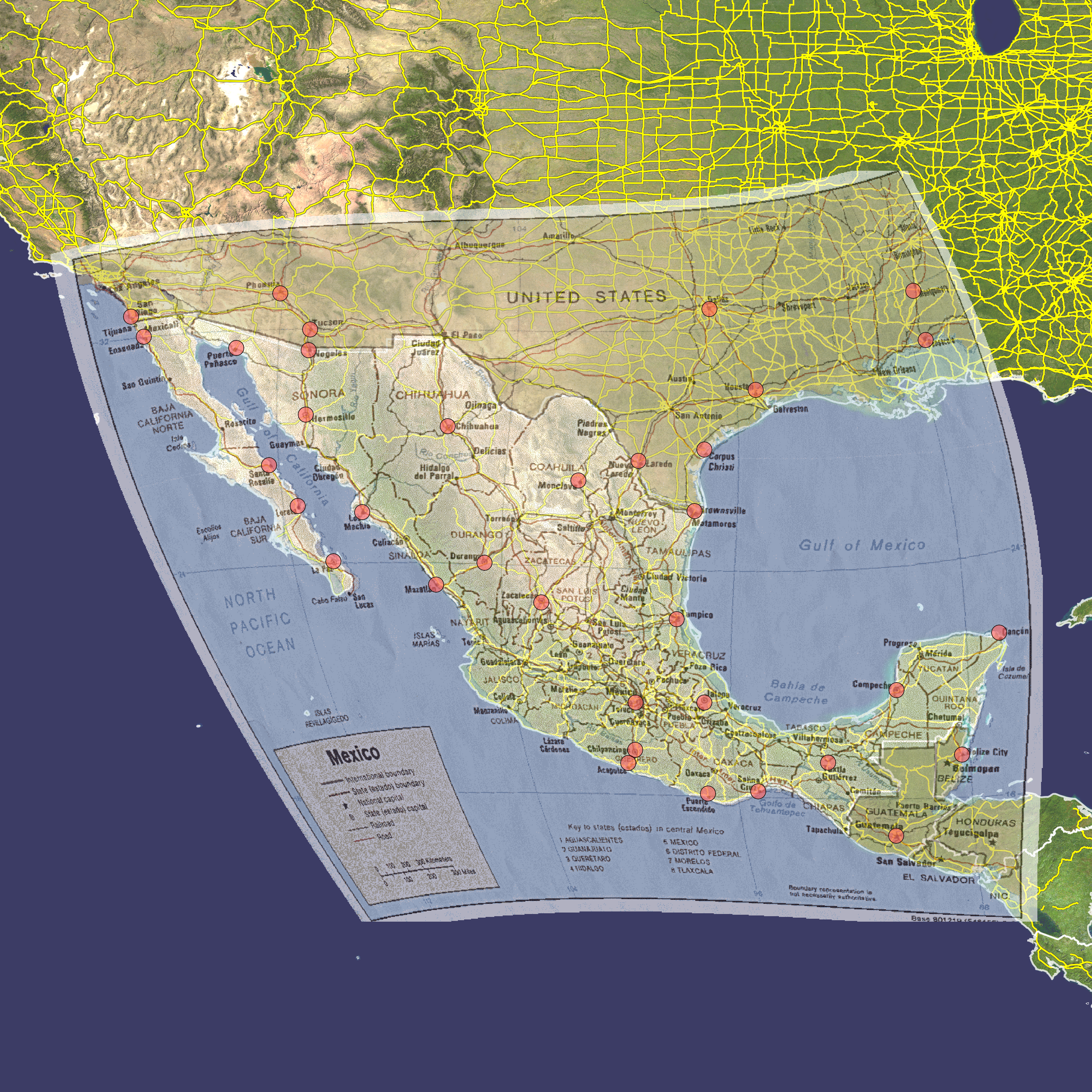

Supplement: S10 Fig — Automatically georeferenced map and control points overlaid on satellite imagery. Map resolution = 1248 x 1010 pixels. ModelMaxLOO = 64.3 pixels (8.0% of image radius). The map image is from the University of Texas at Austin’s Perry-Castañeda Library (PCL) Map Collection and is in the public domain: https://legacy.lib.utexas.edu/maps/americas/mexico.gif. The background satellite data is from NASA Visible Earth’s “Blue Marble” true-color global image mosaic and is in the public domain. The geodata used to render country outlines (in white) and roads (in yellow) is from ©Natural Earth data and is in the public domain. (PNG) [file pone.0260039.s010.png]
